# Tracking antimicrobial resistance transmission in urban and rural communities in Bangladesh: a One Health study of genomic diversity of ESBL-producing and carbapenem-resistant *Escherichia coli*

**DOI:** 10.1128/spectrum.03956-23

**Published:** 2024-05-03

**Authors:** Brandon M. Flatgard, Alexander D. Williams, Mohammed Badrul Amin, Jon L. Hobman, Dov J. Stekel, Emily K. Rousham, Mohammad Aminul Islam

**Affiliations:** 1 Paul G. Allen School for Global Health, Washington State University, Pullman, Washington, USA; 2 Laboratory of Data Discovery for Health Ltd, Hong Kong Science and Technology Park, Tai Po, Hong Kong, China; 3 School of Public Health, University of Hong Kong, Pok Fu Lam, Hong Kong, China; 4 Laboratory of Food Safety and One Health, icddr,b, Dhaka, Bangladesh; 5 School of Biosciences, University of Nottingham, Sutton Bonington Campus, Sutton Bonington, Leicestershire, United Kingdom; 6 Department of Mathematics and Applied Mathematics, University of Johannesburg, Johannesburg, South Africa; 7 School of Sport, Exercise and Health Sciences, Loughborough University, Loughborough, United Kingdom; Emory University School of Medicine, Atlanta, Georgia, USA

**Keywords:** ESBL-producing *E. coli*, whole genome sequencing, antibiotic resistance genes, clonal spread, wastewater

## Abstract

Antimicrobial resistance (AMR) poses a significant threat to global health and sustainable development goals, especially in low- and middle-income countries (LMICs). This study aimed to understand the transmission of AMR between poultry, humans, and the environment in Bangladesh using a One Health approach. We analyzed the whole genome sequences (WGS) of 117 extended-spectrum β-lactamase-producing *Escherichia coli* (ESBL-Ec) isolates, with 46 being carbapenem resistant. These isolates were obtained from human (*n* = 20) and poultry feces (*n* = 12), as well as proximal environments (wastewater) (*n* = 85) of three different study sites, including rural households (*n* = 48), rural poultry farms (*n* = 20), and urban wet markets (*n* = 49). The WGS of ESBL-Ec isolates were compared with 58 clinical isolates from global databases. No significant differences in antibiotic resistance genes (ARGs) were observed in ESBL-Ec isolated from humans with and without exposure to poultry. Environmental isolates showed higher ARG diversity than human and poultry isolates. No clonal transmission between poultry and human isolates was found, but wastewater was a reservoir for ESBL-Ec for both. Except for one human isolate, all ESBL-Ec isolates were distinct from clinical isolates. Most isolates (77.8%) carried at least one plasmid replicon type, with IncFII being the most prevalent. IncFIA was predominant in human isolates, while IncFII, Col(MG828), and p0111 were common in poultry. We observed putative sharing of ARG-carrying plasmids among isolates, mainly from wastewater. However, in most cases, bacterial isolates sharing plasmids were also clonally related, suggesting clonal spread was more probable than just plasmid transfer.

**IMPORTANCE:**

Our study underscores that wastewater discharged from households and wet markets carries antibiotic-resistant organisms from both human and animal sources. Thus, direct disposal of wastewater into the environment not only threatens human health but also endangers food safety by facilitating the spread of antimicrobial resistance (AMR) to surface water, crops, vegetables, and subsequently to food-producing animals. In regions with intensive poultry production heavily reliant on the prophylactic use of antibiotics, compounded by inadequate waste management systems, such as Bangladesh, the ramifications are particularly pronounced. Wastewater serves as a pivotal juncture for the dissemination of antibiotic-resistant organisms and functions as a pathway through which strains of human and animal origin can infiltrate the environment and potentially colonize new hosts. Further research is needed to thoroughly characterize wastewater isolates/populations and understand their potential impact on interconnected environments, communities, and wildlife.

## INTRODUCTION

Antimicrobial resistance (AMR) is a One Health problem that substantially threatens global health and sustainable development goals. Low- and middle-income countries (LMICs) are disproportionately affected by AMR (1, 2). The dynamic interactions between humans and domestic livestock, along with their shared environments, likely serve as important routes for human exposure to antibiotic-resistant pathogens, especially in a setting with inadequate sanitation infrastructure, limited access to safe water, and poor hygienic practices (3).

Raising domestic animals within the same household premises is a common practice in low-resource settings, especially in rural areas where livestock is one of the significant sources of income for households (4–6). In rural Bangladesh, small- and medium-scale poultry farms (300–5,000 birds) are typically in close proximity to households, and waste generated from farms is mixed with household waste and disposed of regularly in the environment, such as surface water bodies (7, 8). There is a general conception among many people in Bangladesh that feces of domestic animals are not harmful to human health, and thus, these are often used for multiple purposes, such as fish feed or fertilizer, which may lead to increased risk of human exposure (8).

In our previous studies, we found that most antibiotics commonly used in small-scale poultry farms in Bangladesh for prophylactic or therapeutic purposes are the same antibiotics used in humans, such as sulfonamides, fluoroquinolones, metronidazole, penicillin, etc. (9). Therefore, the key concern is that antibiotic resistance genes (ARGs) emerging in bacterial pathogens in animals might pose a risk for human health. Although the exact bacterial strains may not always colonize or infect both humans and animals, the sharing of ARGs in a common environment can lead to the ineffectiveness of antibiotics used in human medicine, affecting both commensals and pathogens. In Bangladesh, our previous study found that around 70% of people in a rural community were colonized by extended-spectrum β-lactamase-producing *Escherichia coli* (ESBL-Ec) isolates (10). Similar carriage rates were found in poultry raised with antibiotics and among backyard poultry that scavenge household waste for food (10). We also found that the proximate environments, especially wastewater, pond water, and river water, were highly contaminated with ESBL-Ec (10). However, we do not know whether these ESBL-Ec strains are shared between human and animal hosts in the overlapping environmental landscape.

It is essential to analyze the isolates from a connected environmental landscape to understand the complete picture of transmission dynamics of AMR within and beyond household units in the community. A recent study in Kenya has shown that the sharing of antibiotic-resistant *E. coli* occurs less frequently between humans and animals, and in most cases, it occurs between hosts within the same household unit (11). By examining the core genomes of *E. coli* samples from 99 urban households, this study reported that the genetic variation and distribution patterns of *E. coli* were predominantly influenced by the specific household and were significantly determined by host type (11).

Only a few studies have assessed all three domains of the One Health paradigm (human, animal, and environment) for AMR surveillance, especially in LMICs (12). Besides, studies with systematically collected samples from humans, animals, and environments connected on temporal and spatial scales are lacking. Genomic fingerprinting of bacterial isolates from all three domains to investigate clonal relationships is fundamental to transmission studies, and the characterization of isolates using whole genome sequencing (WGS) has increasingly been used for this purpose. Although data from LMICs are lacking, antibiotic-resistant Enterobacterales, particularly ESBL-producing Enterobacterales (ESBL-E) and carbapenem-resistant Enterobacterales (CR-E)—the first priority critical group of bacteria according to WHO—are associated with a significant burden of community- and healthcare-associated infection (13, 14). Both ESBL-E and CR-E confer resistance to common antibiotics, and occasionally, they become resistant to all available antibiotics. For this reason, the US-CDC considers these two types of drug resistance as serious and urgent threats because of the limited treatment options (15).

The primary aim of this study was to investigate the clonal overlaps (CO) of AMR organisms between poultry, humans, and their shared environments. We considered frequent exposure to poultry as a risk factor for human colonization with AMR organisms originating from poultry. Using WGS analysis, we assessed the phylogenetic relationship of the ESBL-Ec and carbapenem-resistant *E. coli* (CR-Ec) isolates across rural and urban landscapes in Bangladesh obtained from our previous study (10).

## MATERIALS AND METHODS

### Selection of antibiotic-resistant *E. coli* isolates for phylogenetic analysis

We had previously conducted a point prevalence study using an integrated approach to estimate the prevalence and abundance of ESBL-Ec, CR-Ec, and their associated resistant genes in different environmental compartments that are likely to be impacted by intensive poultry production/selling practices in both urban and rural areas of Bangladesh (10). The comprehensive study explored the carriage of ESBL-Ec in adults across three different settings with close interactions between humans and poultry, including rural households with backyard poultry, small commercial broiler poultry farms in rural settings, and urban wet markets where poultry is sold, slaughtered, and processed on site. In each environment, the study selected adults who had daily contact with poultry (categorized as high exposure) and a comparison group from the same environment who had little to no direct contact with poultry (categorized as low exposure) yet lived in conditions that were otherwise similar. The transmission of AMR was assessed through geospatial mapping of the abundance of ESBL-Ec and corresponding resistance genes in drinking water supply, wastewater, surface water (pond and river water), and solid waste proximal to each site (16). Fecal samples from humans and poultry were contemporaneously collected. The sampling took place at two time points to capture the prevalence in two seasons (winter and summer). This study also identified potential behaviors relating to human–poultry interactions in each location that contribute to AMR transmission (7, 9). The conceptual framework has been outlined previously (3). Briefly, we collected samples from 20 rural villages, 40 commercial broiler farms in Mirzapur Upazilla of Tangail district, and 40 urban wet markets in Dhaka city. In every village, farm, and market, we collected samples from one adult who had significant contact with poultry, one adult with minimal contact with poultry, and one chicken linked to the aforementioned human with high exposure. The drinking water supply and wastewater outlet for each household/farm/market were also sampled. From rural areas, we sampled pond water located within the same property and river water at the closest point to the property. The ponds collect runoff from household drains, drainage ditches, and rainwater/surface water. All samples were tested for ESBL-Ec and CR-Ec by directly culturing samples on CHROMagar ESBL and CHROMagar CARBA plates (CHROMagar, Paris, France), as described previously (3). Characteristic colonies on these plates were counted. Two well-isolated colonies from each selective plate representative of each sample were confirmed as *E. coli* using standard biochemical tests and were stored in glycerol broth at −80°C prior to further characterization. In our previous studies, we also tested one ESBL-Ec isolate from a given sample for antibiotic susceptibility against 16 antibiotics by standard disc diffusion method following Clinical and Laboratory Standards Institute (CLSI) guidelines (10, 16).

### Whole genome sequencing of antibiotic-resistant *E. coli* isolates

A total of 117 ESBL-Ec isolates, including 46 CR-Ec, were selected for WGS and bioinformatics analyses. To capture the diversity of isolates within and between household/farm/market units, we selected isolates from human, poultry, and wastewater samples within each unit and solid waste, as well as samples from ponds and rivers , categorized into winter and summer seasons. We selected one isolate from a given sample (Table 1).

**TABLE 1:** Distribution of samples used for selection of ESBL-Ec and CR-Ec for WGS analysis^*a*^

Study sites	No. of unit	No. of isolates	Types of samples								
			Poultry (*n* = 12)	Environment (*n* = 85)							Human (*n* = 20)
			PC	PW	RW	PDW	WW	PP	AM	SW	HF
Households	24	48	2	6	17	1	8	3	4	0	7
Farms	7	20	5	2	5	0	4	0	0	0	4
Wet markets	17	49	5	0	0	1	19	1	0	14	9
Total	48	117	12	8	22	2	31	4	4	14	20

^*a*^ PC, poultry cloacae; PW, pond water; RW, river water; PDW, poultry drinking water; WW, wastewater; PP, poultry pens; AM, animal manure; SW, solid waste; HF, human feces.

### DNA extraction and whole genome sequencing

DNA was extracted from an overnight culture of *E. coli* isolates following the procedure described earlier using the Maxwell culture DNA extraction kit and Maxwell automated nucleic acid extraction system (Promega, Madison, WI, USA) following the manufacturer’s instructions (17). The purity and concentration of the extracted DNA were evaluated using a NanoDrop spectrophotometer (Thermo Fisher Scientific, USA) and Qubit 2.0 fluorometer (Life Technologies, Carlsbad, CA, USA), respectively. Libraries were prepared using the Nextera XT kit, and 150 bp paired-end sequencing was performed using the Illumina NextSeq500 platform (Illumina, San Diego, CA, USA). FastQC v0.11.4 was used to assess the reading quality (18).

### Bioinformatic analyses

#### Analysis of WGS of isolates for ARGs, virulence genes, plasmid replicon types, phylogroups, and mobile genetic elements (MGEs)

Paired raw reads were trimmed using CLC Genomics Workbench v21.0.4 (https://digitalinsights.qiagen.com/) with a quality limit of 0.05, ambiguous nucleotides (max = 2), maximum length of 150 nucleotides, and a minimum length of 50 nucleotides. Virulence genes were identified using quality control (QC) raw reads with VirulenceFinder v2.0, database version (2020-05-29), with minimum coverage and identity of 90% (19–21). Resistance genes were identified using QC raw paired reads with the ResFinder v4.1 database (2022-02-04) and PointFinder database (2021-02-01) with minimum coverage and identity of 90% (19, 22, 23). Plasmid genes were identified using PlasmidFinder v2.0.1 database (2021-11-29) with a minimum coverage and identity of 90% (19, 24). ClermonTyping was used for *E. coli* phylotyping (25). Achtman multi-locus sequence typing (MLST), phylotype, O- and H-antigen types for each isolate were identified using EnteroBase (26). Heatmaps were generated using R-package *ComplexHeatmap* v2.10.0 using binary distances (27). Mobile genetic elements were predicted using MGEFinder v1.0.6 (28). The prevalence of resistance genes, virulence genes, plasmid replicon types, and other accessory genes among isolates between study groups was compared using Fisher’s exact tests in IBM SPSS Statistics v27 (29). The Kruskal-Wallis test was performed using the R-package *ggstatsplot* with Bonferroni correction to adjust for multiple comparisons*,* and permutational multivariate analysis of variance (PERMANOVA) was performed using the *adonis2* function of the R-package *vegan* (30, 31). The permutation-based *P* values were determined using 9,999 permutations.

#### Phylogenetic analyses of isolates from different sources and study sites

A single nucleotide polymorphism (SNP) analysis of the core genome was constructed using previously trimmed reads and assembled using Unicycler v0.5.0 with options “--min_fasta_length 400” (32). The resulting contigs were annotated using Bakta v1.7.0 with options “--genus Escherichia --species coli” (33). The pangenome was calculated using Roary v3.13.0 with options “-e -n” (34). The SNPs were extracted from the resulting multi-fasta alignment using SNP-sites v2.5.1 with options “-r -m -v -p” (35). A tree was generated from the extracted SNPs using IQtree v2.2.0.3 with bootstrapping set to 1,000 (option “-B 1000”) and the standard model of substitution (option “-m GTR+F+G4”) (36). The resulting SNP tree was visualized with iTOL v5 (37). We considered two isolates to be clonal if they differ from each other by <22 SNPs, according to previous studies regarding *Klebsiella pneumoniae* (38). To further analyze clustering, EnteroBase was used to calculate cgMLST v1 + HierCC v1:201423, generate a minimum spanning tree (MSTreeV2), and visualize a GrapeTree with <11 allelic differences between isolates being considered as clonal (26, 39).

#### Identification of putative bacterial sharing

Filtered raw reads were assembled using Unicycler v0.5.0 with options “--min fasta length 400” (32). Plasmid contigs were grouped into distinct clusters and partially reconstructed using MOB-suite v3.1.0 with mob-recon module (40, 41). To find the location of resistance genes (plasmid or chromosome), the partially reconstructed chromosome and plasmid output was run through ABRicate v1.0.1 with ResFinder v4.1 database by setting a cutoff for resistance gene identity and coverage at ≥90% (19, 22, 23, 42). Sharing was determined by calculating the MASH pairwise distance using the Pangenome Analysis Toolkit v1.0.6 (PATO) package in R (43). The whole-genome average nucleotide identity (ANI) was calculated as ANI = 1 – Distance_MASH_ × 100. Sharing was only considered when pairs had an ANI above 99.9% (44). Sharing was analyzed among different sources (poultry, human, environment), within and between locations (rural households, rural poultry farms, and urban wet markets). Sharing was calculated for the reconstructed plasmids and chromosomes separately.

#### Comparative genomic analysis of isolates with clinical strains of the same sequence types (ST) available in global databases

Clinical strains were selected from the NCBI Isolate Browser (https://www.ncbi.nlm.nih.gov/pathogens/isolates/) and EnteroBase, including only *E. coli* clinical isolates obtained from human patients in Bangladesh and elsewhere. Additionally, the isolate search focused on the predominant Achtman MLST schemes represented in this study. We aimed to include at least 10 clinical isolates of each dominant STs, but due to the lack of a sufficient number of complete genomes in GenBank, this was not possible. Altogether, 58 clinical isolates were included in the comparative analysis originating from patients from different parts of the world, 18 of which originated from Bangladesh. The complete list of clinical isolates used in this study can be found in the supplementary materials (Table S1).

#### Complete sequencing of plasmids by long-read sequencing approach

To characterize the plasmids, we selected two *E. coli* isolates that were ESBL producing and carbapenem resistant, one each from the urban and rural areas, for long-read sequencing. One isolate originated from downstream river water (rural) (TR-110-DRW-K1), and the other from solid waste from an urban wet market (DL-185-SS2-K1). Both isolates were resistant to all 16 antibiotics tested in the study except colistin and were positive for multiple plasmids based on short-read sequencing data. Long-read sequencing libraries were prepared by Novogene Co., Ltd, using the PacBio single-molecule real-time (SMRT) Sequel system (Pacific Biosciences California, Inc.). Although *de novo* assembly of long-read sequences often produces highly contiguous and complete prokaryotic genomes, it has been acknowledged that no single method is perfect, and each is likely to possess contrasting strengths and weaknesses (45, 46). Accordingly, a consensus approach based on multiple assemblies has been suggested to improve final assembly output (46, 47). In the present study, we, therefore, attempted to generate preliminary assemblies for each of the isolates sequenced using different assembly pipelines for long reads, including Canu v2.1.1 (48), Flye v2.8.3-b1701 (49), wtdbg2 v2.5 (50), and Raven v1.5.0 (51). In addition, a hybrid assembly of short and long reads (Illumina and PacBio, respectively) was produced using Unicycler v0.4.9b in hybrid mode (52). The output of these different assembly methods was then compared using QUAST v5.0.2 (53). Finally, the best assembly for each isolate was chosen based on the consensus produced by Trycycler v0.4.1 (47) or the most contiguous assembly, where a consensus could not be achieved. Assemblies were polished using gcpp v2.0.2 from PacBio Tools (https://github.com/PacificBiosciences/gcpp). For isolate DL-185-SS2-K1, the Raven assembly was used, while for TR-110-DRW-K1, the Trycycler consensus assembly was selected. Polished genomes were annotated using Prokka v1.14.6 (54). Plasmids were further characterized using the online web server for PlasmidFinder v2.0.1 database (2021-01-13) (24). Plasmid maps were produced using SnapGene. Plasmid map annotations were manually curated by cross-referencing the Prokka annotations with UniProt BLASTp results (https://www.uniprot.org/) in addition to CARD BLASTp for antibiotic resistance genes (ARG identity and coverage ≥90%) (https://card.mcmaster.ca/).

## RESULTS

### Selection of *E. coli* for WGS analysis using a One Health approach

A total of 117 ESBL-Ec/CR-Ec were sequenced in this study, of which 20 were obtained from humans, 12 from poultry, and 85 from environmental sources (Table 1). Of the 117 isolates, 48 were from rural households, 20 were from rural poultry farms, and 49 were from urban wet markets. In addition, we included WGS data of 58 clinical isolates available in the GenBank to compare with the 117 study isolates.

### Analysis of ESBL-Ec and CR-Ec genomes for ARGs and their spatial distribution

All isolates were positive for ARGs for multiple antibiotics. There were some differences in ARG profiles based on the carbapenem susceptibility status of the isolates. Around 76% (54/71) of the carbapenem-sensitive ESBL-Ec isolates were positive for quinolone resistance genes, followed by 55% (39/71) for tetracycline resistance genes. Concerning all isolates, among β-lactamase genes, isolates from all three sources were predominantly positive for *bla*_CTX-M-15_ (76.1% [89/117]), followed by *bla*_TEM-1b_ (56.4% [66/117]) and *bla*_OXA-1_ (25.8% [30/117]). The carbapenem resistance genes found in CR-Ec isolates were only *bla*_NDM_ type, including *bla*_NDM-5_ (73.9% [34/46]), *bla*_NDM-7_ (15.2% [7/46]), and *bla*_NDM-1_ (8.7% [4/46]). Two isolates were negative for *bla*_NDM_ type gene. High proportions of CR-Ec isolates were positive for aminoglycoside (93% [43/46]), trimethoprim (85% [39/46]), sulfonamide (83% [38/46]), macrolide (78% [36/46]), phenicol (65% [30/46]), and tetracycline (63% [29/46]) resistance genes (Table S2 and Fig. S1, full gene breakdown Table S3). Although virulence genes were cataloged for each strain, a detailed analysis was not performed. The distribution of the top 15 virulence genes has been presented in Figure S1.

The prevalence of ARGs varied according to the source of the isolates. Environmental isolates exhibited a greater diversity of ARGs than those from human and poultry sources. A significantly higher proportion of poultry isolates (8/12) was positive for tetracycline resistance genes (Table 2; Table S3). Quinolone resistance genes were more prevalent in human (17/20) and environmental isolates than in poultry isolates (8/12). Notably, colistin, fosfomycin, and rifampicin resistance genes were found only in environmental isolates (Table 2).

**TABLE 2:** Distribution of antibiotic resistance genes among ESBL-Ec isolates from human, animal, and environmental sources

ARGs related to antibiotic class	% of isolates positive			*P* value
	Poultry (*n* = 12)	Environment (*n* = 85)	Human (*n* = 20)	
Aminoglycoside	8.3	16.5	5.00	0.505
β-lactam	100	100	100	NA^*a*^
Carbapenem	25.0	23.5	25.0	1.000
Colistin	0.00	4.7	0.00	0.458
Fluroquinolone	8.3	1.2	0.0	0.223
Fosfomycin	0.00	2.4	0.00	1.000
Macrolide	50.0	49.4	40.0	0.793
Phenicol	33.3	40.0	20.0	0.237
Quinolone	66.7	80.0	85.0	0.458
Rifampicin	0	4.7	0.0	1.000
Sulfonamide	58.3	55.3	30.0	0.115
Tetracycline	75.0	61.2	35.0	**0.048^*b*^**
Trimethoprim	66.7	56.5	30.0	0.057

^*a*^ NA, not applicable.

^*b*^ Bold values indicate statistical significance using Fisher's exact test.

**Fig 1:**
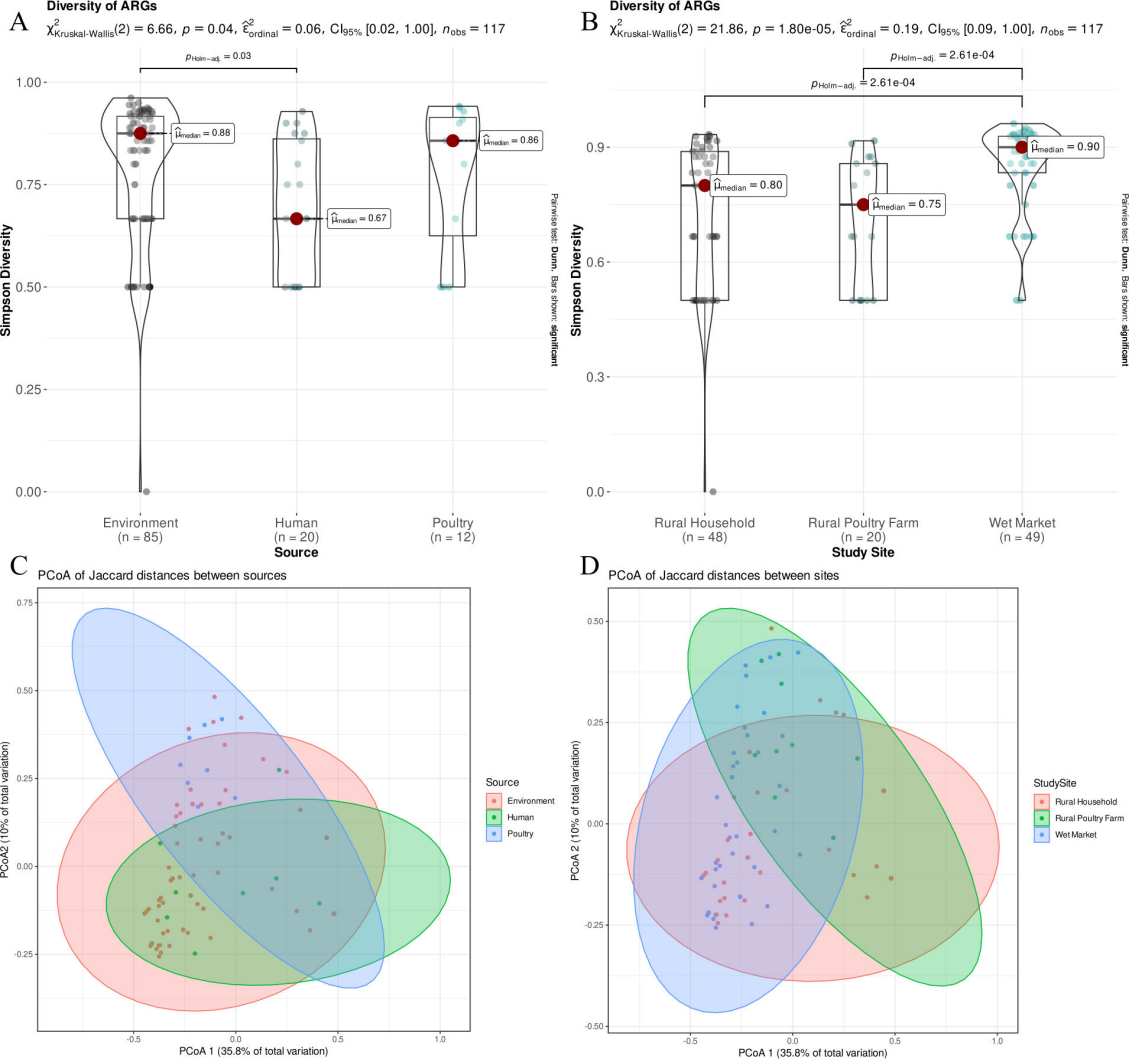
Statistical tests showing the difference in diversity of ARGs between isolates from three different sources (human, poultry, and environment) and study sites (urban wet markets, rural households, and rural poultry farms). Violin plots comparing the ARG diversity among isolates from different (**A**) sources and (**B**) study sites through Kruskal-Wallis test and Dunn pairwise test. Principal coordinate analysis (PCoA) of Jaccard distances of ARGs between isolates from different (**C**) sources and (**D**) study sites.

The dissimilarity in the distribution of ARGs was highest between human and environmental sources, with a Bonferroni-adjusted *P* value of 0.04 (Fig. 1A). Using the same method, the diversity of ARGs among the study sites was compared. The dissimilarity in the distribution of ARGs was observed to be highest between the urban wet markets and rural households, as well as between the urban wet markets and rural poultry farms (Bonferroni-adjusted *P* value < 0.001) (Fig. 1B). By comparing only wastewater isolates from the three study sites, we observed that the isolates from the urban wet market had a higher dissimilarity in ARG distribution than the isolates from both rural households and rural poultry farms with a Bonferroni-adjusted *P* value = 0.02 (Fig. S2).

A PERMANOVA analysis was performed to test the differences in community diversity of ARGs between isolates from three sources (human, poultry, and environment). The results showed a marginal effect of source on ARG diversity (F = 2.04, *P* = 0.05), indicating that the ARG diversity differed significantly between the three sources. However, the model only accounted for 3% of the overall variation in the data, as indicated by an R-squared value of 0.03 (Fig. 1C). Applying the same methodology, we conducted a comparison of the study sites and found a significant effect of study sites on the diversity of ARG (F = 4.32, *P* = 0.01). The analysis suggested that there were notable variations in ARG diversity among the three study sites. The model accounted for 7% of the overall variation in the data (R-squared = 0.07) (Fig. 1D).

### Diversity of β-lactamase genes in ESBL-Ec isolates from human, poultry, and environmental sources

A total of 33 subtypes of β-lactamase genes were identified among 117 isolates, with the highest diversity being observed in isolates from poultry sources. As mentioned previously, only three were commonly found among isolates from all three sources, of which *bla*_CTX-M-15_ was the predominant gene, followed by *bla*_TEM-1b_ and *bla*_OXA-1_. There were no commonalities in other β-lactamase genes between human and poultry isolates (Table 3).

**TABLE 3:** Carriage of the common β-lactamase genes in either chromosome or plasmid among ESBL-Ec isolates from all three sources

β-Lactamase gene types	Poultry (*n* = 12)		Human (*n* = 20)		Environment (*n* = 85)	
	Chromosome	Plasmid	Chromosome	Plasmid	Chromosome	Plasmid
*bla*_CTX-M-15_	4	2	13	6	32	30
*bla*_OXA-1_	1	1	3	1	13	11
*bla*_TEM-1b_	0	7	1	5	6	35
Total	5	10	17	12	51	76

Of the 20 human isolates, only five subtypes of β-lactamase genes were detected, whereas 15 subtypes were identified in 12 poultry isolates and 24 subtypes in the 85 environmental isolates (Table S3). *AmpC* β-lactamase genes (*bla*_CMY_ family) were the least common among poultry (0/12) and human (1/20) isolates, with a higher prevalence in environmental (15/85) isolates. The *bla*_CTX-M-15_ gene was detected in over 95% (19/20) of human isolates, whereas 50% (6/12) of poultry and 75% (64/85) of environmental isolates were positive for this gene. Poultry isolates exhibited higher diversity in *bla*_TEM_ subtypes, with the detection of 9 out of 12 subtypes, while human isolates were only positive for the subtype *bla*_TEM-1b_. β-lactamase genes were found in the chromosome of 75% (15/20) of human isolates, 59% (50/85) of environmental isolates, and 58% (7/12) of poultry isolates.

### Phylogroup, sequence type, and plasmid replicon type of ESBL-Ec and CR-Ec isolates

Based on the Clermont typing scheme, all 117 isolates were grouped into eight phylogroups. The most prevalent phylogroup was B1 (38.5%), followed by phylogroup A (26.5%), D (22.2%), F (5.1%), C (4.3%), B2 (1.7%), E (0.9%), and G (0.9%). Phylogroup B1 was the most common among isolates from all three sources. Environmental isolates were associated with phylogroup common to both animals and humans. Phylogroup B2, which is clinically most important, was not found among poultry isolates, while only one human isolate and one environmental isolate belonged to this phylogroup. A full phylogroup and ST breakdown is listed in Table S4.

Based on seven Achtman housekeeping genes, 117 *E. coli* isolates were grouped into 71 STs, of which ST155 was the most prevalent, accounting for more than 12% (*n* = 14) of the isolates from all three sources. Maximum diversity of STs was found among isolates from humans, with 19 STs among 20 isolates (95% variability), followed by poultry with 10 STs among 12 isolates (83% variability) and the environment with a total of 42 STs in 85 isolates (49% variability). Of the 71 STs, only ST155 and ST206 were common between human and poultry isolates, and ST155 was also identified among environmental isolates. Interestingly, 11 out of 19 STs (58%) among human isolates overlapped with environmental isolates. In the case of poultry, 2 out of 10 STs (20%) overlapped with environmental isolates, and the frequency of sharing was more common among isolates belonging to ST405 (Table 4). Overall, the sharing of isolates between humans and poultry was notably less frequent, while overlapping of environmental isolates with either human isolates or poultry isolates was more frequent (Table 5).

**TABLE 4:** Number of STs belonging to ESBL-Ec isolates from multiple sources^*a*^

Total no. of ST (*n* = 71)	No. of ST overlapping between sources		
	Human	Poultry	Environment
Human (no. of ST = 19)	NA	2	11
Poultry (no. of ST = 10)	2	NA	2
Environment (no. of ST = 42)	11	2	NA

^*a*^ NA, not applicable.

**TABLE 5:** CO of ESBL-Ec isolates from different sources and study sites

CO	Sample ID	Location	Source	ST	Serotype
Between locations (household [HH] and wet market [WM])					
1	TR-011-DRW	HH	River water	405	O102:H6
	DL-062-SS1	WM	Solid waste	405	O102:H6
	DL-165-SS1	HH	Solid waste	405	O102:H6
	DL-064-WW	WM	Wastewater	405	O102:H6
2	DL-187-SS1	WM	Solid waste	405	O102:H6
	DL-183-SS1	WM	Solid waste	405	O102:H6
	TR-019-FPW	HH	Fresh pond water	405	O102:H6
	TR-104-DRW	HH	River water	405	O102:H6
3	DL-083-SS2	WM	Solid waste	8346	O64:H10
	TR-110-DRW	HH	River water	8346	O64:H10
4	TR-21-DRW	HH	River water	6683	O18:H49
	DL-67–01-FH	WM	Human high exp	6683	O18:H49
Between locations (household [HH] and rural farm [RF])					
5	TF-122-DRW	RF	River water	38	O60:H30
	TR-108-DRW	HH	River water	38	O60:H30
	TR-119-DRW	HH	River water	38	O60:H30
6	TR-109-AM	HH	Animal manure	155	O-:H25
	TR-109–01-FH	HH	Human high exp	155	O-:H25
	TF-125-DPW	PF	Pond water	155	O-:H25
	TF-125-WW	PF	Wastewater	155	O-:H25
	TR-05-FPW	HH	Fresh pond water	155	O-:H25
7	TF-125–01-FH	PF	Human high exp	131	O25:H4
	TR-05-DRW	HH	River water	131	O25:H4
	SRR11496578	Global	Clinical	131	O25:H4
Same location (different wet markets [WM])					
8	DL-182-FP1	WM	Poultry pens	405	O102:H6
	DL-182-CL	WM	Chicken cloacae	405	O102:H6
	DL-167-WW2	WM	Wastewater	405	O102:H6
9	DL-182–02-FH	WM	Human high exp	68	O99:H6
	DL-185-SS2	WM	Solid waste	68	O99:H6
10	DL-180–02-FL	WM	Human low exp	448	O1:H19
	DL-181-WW	WM	Wastewater	448	O1:H19
	DL-187-WW	WM	Wastewater	448	O1:H19
11	DL-185-WW	WM	Wastewater	4450	O53:H18
	DL-169-WW	WM	Wastewater	4450	O53:H18
Same location (different households [HH])					
12	TR-113-DRW	HH	River water	101	O160:H31
	TR-111-DRW	HH	River water	101	O160:H31
	TR-101-DRW	HH	River water	101	O160:H31
	TR-102-DRW	HH	River water	101	O160:H31
Same location (same rural farm [RF])					
13	TF-130-CL	RF	Chicken cloacae	155	O153:H9
	TF-130-DRW	RF	River water	155	O153:H9
Same location (same wet market [WM])					
14	DL-069-SS	WM	Solid waste	361	O9:H30
	DL-069-WW	WM	Wastewater	361	O9:H30
Same location (same household [HH])					
15	TR-10-FP	HH	Poultry pens	1727	O-:H7
	TR-10-AM	HH	Animal manure	1727	O-:H7
	TR-10-FPW	HH	Pond water	1727	O-:H7
16	TR-10-WW	HH	Wastewater	3489	O147:H40
	TR-10-DRW	HH	River water	3489	O147:H40

Analysis of plasmid replicon types among isolates revealed that 78% (91/117) of the isolates carried at least one plasmid (Table 6; Fig. S1). Plasmid carriage was predominant among poultry isolates (83%, 10/12), followed by environmental (80%, 68/85) and human isolates (65%, 13/20) (Table 6; Fig. S1). IncFII was the most prevalent plasmid type (10.5%, *n* = 42), followed by IncFIA (10%, *n* = 40), IncFIB (AP001918) (9.5%, *n* = 38), IncI (4.8%, *n* = 19), and P0111 (4.8% *n* = 19) (Table 5). The presence of IncFIA was most common among human isolates, while P0111, IncFII, and Col440I were common among poultry isolates (Table 6; Fig. S1). Environmental isolates shared commonality with human and poultry isolates, with IncFII, IncFIA, and IncFIB (AP001918) being the common plasmid replicon types (Table 6; Fig. S1). The same plasmid types were also identified in ESBL-Ec and CR-Ec isolates from different sources. IncFIB (AP001918) was identified in 25% (3/12) of poultry, 25% (5/20) of human, and 35% (30/85) of environmental isolates, of which 65.8% (25/38) of the isolates were CR-Ec while the remaining were ESBL-Ec. The presence of plasmid types was also associated with the ARG patterns of isolates. We found that isolates carrying multiple plasmid replicon types were positive for a significantly higher number of ARG types than isolates positive for a single or no plasmid (*P* ≤ 0.001) (Fig. S3). Plasmid replicon types were more abundant in environmental isolates (3.6/isolate) than in poultry (3.2/isolate) and human isolates (2.4/isolate) (Table 6).

**TABLE 6:** Diversity of plasmid replicon types identified in ESBL-Ec isolates from poultry, human, and environmental sources

Plasmid family	Plasmid type	No. of isolates positive^*a*^		
		Poultry	Human	Environment
		(*n* = 12)	(*n* = 20)	(*n* = 85)
Col	Col(BS512)	1	0	11
	Col(IRGK)	0	0	1
	Col(MG828)	2	0	15
	Col156	0	2	6
	Col440I	2	3	12
	Col440II	0	0	1
	ColKP3	0	0	1
	ColpVC	0	1	5
	ColRNAI	1	3	6
IncA/C	IncA/C2	0	0	1
IncB/O/K/Z	IncB/O/K/Z	0	0	1
IncF	IncFIA	1	8	31
	IncFIA(HI1)	1	0	1
	IncFIB(AP001918)	3	5	30
	IncFIB(K)	2	0	4
	IncFIB(pB171)	2	0	13
	IncFIB(pLF82)	1	0	0
	IncFIC(FII)	2	1	4
	IncFII	2	6	34
	IncFII(29)	0	4	4
	IncFII(pAMA1167-NDM-5)	0	1	10
	IncFII(pCoo)	0	2	2
	IncFII(pHN7A8)	1	2	4
	IncFII(pRSB107)	0	0	7
	IncFII(pSE11)	1	1	6
IncH	IncHI1A	0	0	4
	IncHI1B(CIT)	0	0	3
	IncHI1B(R27)	0	0	2
	IncHI2	1	0	7
	IncHI2A	1	0	5
IncI	IncI	0	3	16
	IncI1	3	2	8
	IncI2	0	1	4
IncN	IncN	3	0	5
	IncN2	0	0	1
IncQ	IncQ1	0	0	2
IncR	IncR	2	0	5
IncX	IncX1	1	0	7
	IncX3	0	1	8
	IncX4	0	0	2
IncY	IncY	2	2	8
p0111	p0111	3	0	16

^*a*^ Isolates carrying multiple plasmid types are included in the table.

### Clonal relationship

**Fig 2:**
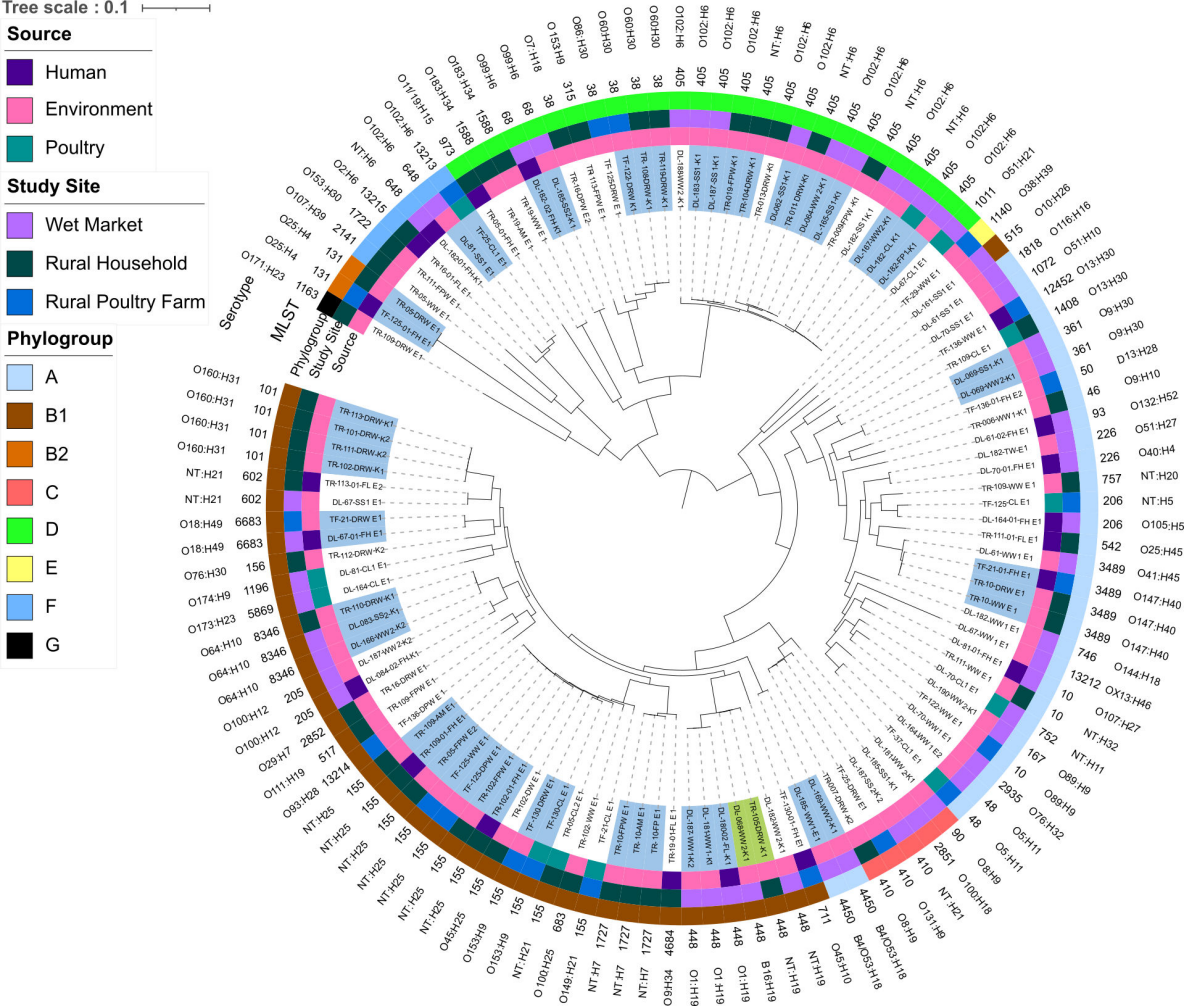
Maximum-likelihood SNP-based phylogenetic tree of 117 ESBL-*E. coli* isolates from humans (*n* = 20), poultry (*n* = 12), and environmental sources (*n* = 85) in various poultry-intensive sites in Bangladesh. The tree was constructed by trimmed reads and was assembled using Unicycler v0.50. The contigs were annotated using Bakta v1.7.0. The pangenome was calculated using Roary v3.13.0. The SNPs were extracted from the resulting multi-fasta alignment using SNP-sites v2.5.1. A tree was generated from the extracted SNPs using IQtree v2.2.0.3 with bootstrapping set to 1,000 and a standard model of substitution. The resulting SNP tree was visualized with iTOL v5. Blue and green highlights indicate a clonal relationship between isolates (<22 SNPs). Colored rings represent source (inner ring), study site (second ring from the center), phylogroup (third ring), MLST (fourth ring), and serotype (fifth ring).

To identify the clonal relationship of ESBL-Ec and CR-Ec isolates between human, animal, and environmental reservoirs in three different sites (rural households, urban markets, and poultry farms), we analyzed core genome-based SNPs and core-genome MLST of the isolates. In these analyses, we included several clinical isolates of the same ST available in the NCBI database to assess whether isolates from non-clinical sources are phylogenetically related to the clinical isolates. We observed at least 16 cases of CO between isolates from different sources or study sites (Fig. 2; Table 5). CO was more evident among isolates belonging to ST405 and ST155. Of the 16 CO cases, four were between isolates from distant locations (urban and rural, ~40 miles apart), three were between isolates from the same areas but different households/farms/markets, and the remaining 9 CO cases were between isolates from different samples of the same study site (Fig. 2; Table 5). The CO was more common among isolates from environmental sources. There were no cases of CO between human and poultry isolates, indicating no direct transmission of organisms between these sources. A few cases of CO were identified between isolates from humans or poultry and the environment, suggesting that the environment is contaminated with antibiotic-resistant organisms from both sources. However, we did not find CO among isolates from all three sources in the study, indicating the lack of exchange of isolates between humans and poultry via exposure to the contaminated environment. Comparison with clinical isolates of matching STs of isolates in this study identified only one case of CO with an isolate from a participant with high exposure to poultry in a rural poultry farm and an isolate obtained from downstream river water in the same rural area. All three isolates belonged to ST131 (O25:H4).

**Fig 3:**
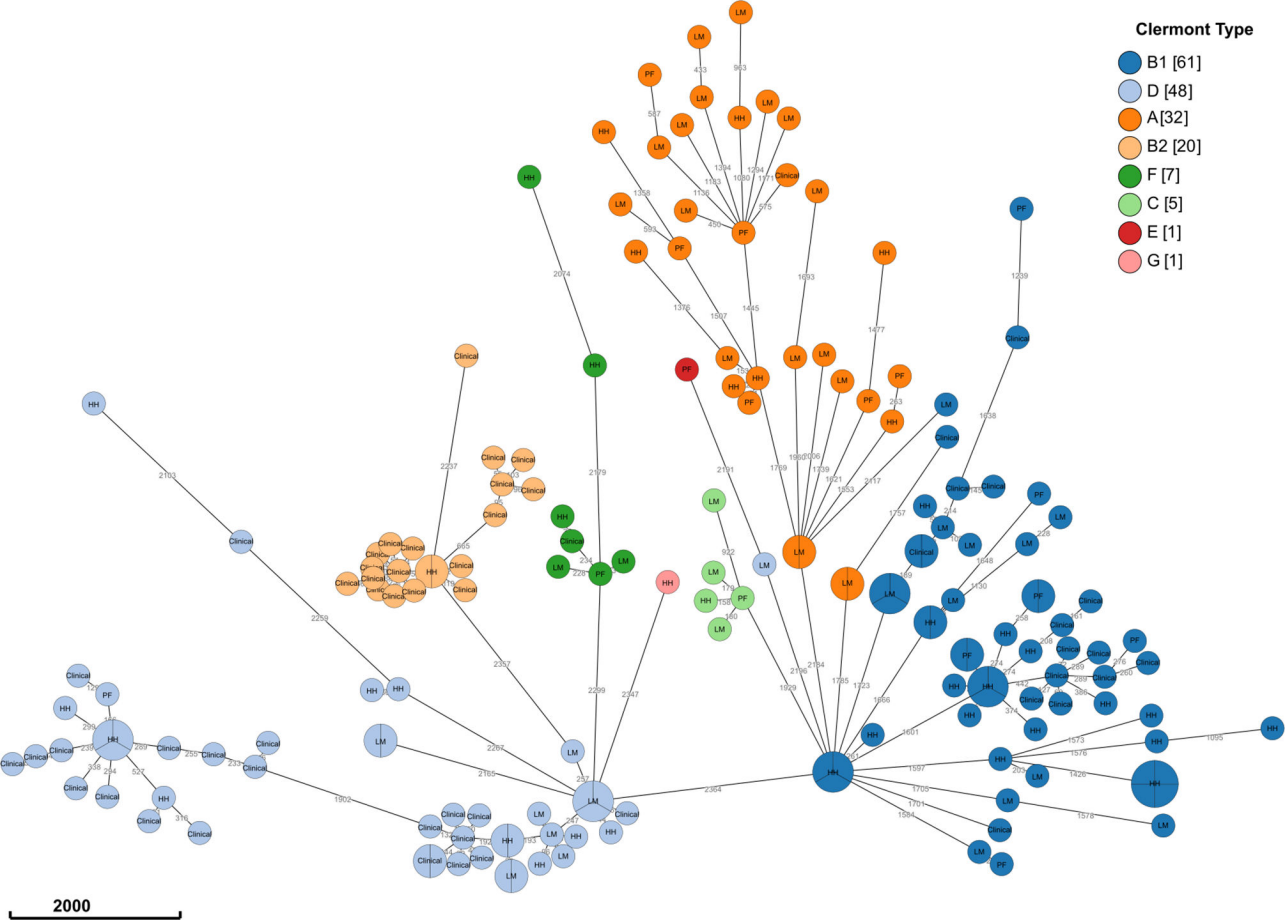
Grapetree displaying an MSTree v2 generated using cgMLST V1 + HierCC V1. Node ID indicates study sites, such as wet markets, rural households, and rural poultry farms (LM, HH, PF, respectively), and clinical isolates. Colors indicate Clermont phylotype. Distance refers to the number of differing alleles. Combined nodes are <11 alleles different.

Core genome SNP analysis of isolates also corresponded to the cgMLST analysis. Considering <11 allelic differences between isolates forming a cluster, we found that out of 117 isolates, 70 were grouped into 23 clusters, while the remaining isolates did not group in a cluster. Nine of these 23 clusters had isolates from multiple sources/study sites, and two clusters had clinical and non-clinical isolates (Fig. 3; Fig. S4).

### ARG plasmid sharing

We also investigated the putative sharing of ARG-carrying plasmids among isolates. Sharing of plasmids was more frequently identified among isolates from wastewater and solid waste in the urban wet market. Of the seven cases with a shared plasmid, four were between isolates within the same stall in the market (Table 7). Sharing of plasmids between isolates from urban and rural areas was only detected in one case. Of the seven shared plasmids, three were IncFIA-type, of which one carried *bla*_NDM-5_, *bla*_CTX-M-15,_ and *bla*_TEM-1b_. The remaining plasmid types were IncIγ/K1 carrying *bla*_CMY-2_, IncX4 carrying *bla*_TEM-1b_*_,_* and IncHI1B carrying multiple antibiotic resistance genes, including *bla*_CTX-M-15_. Interestingly, in six out of seven cases, *E. coli* isolates carrying these plasmids were clonally related based on core genome SNP analysis, indicating that sharing of the plasmid is not an independent event; instead, in most of the cases, it was associated with the clonal overlaps of the strain.

**TABLE 7:** Potential sharing of reconstructed plasmid contigs

Plasmid sharing	Isolate ID	Source	Clonal overlaps	ST	Shared plasmid type	Plasmid carrying AMR genes
1	DL-069-WW2	Wastewater	Yes	361	Untypeable	*aph(3')-Ia, cmlA1, ant(3'')-Ia, sul3, mef(B*)
	DL-069-SS1	Solid waste		361		*aph(3')-Ia, cmlA1, ant(3'')-Ia, sul3, mef(B*)
2	DL-069-WW2	Wastewater	Yes	361	IncFIA	*bla*_TEM-1B_ *, dfrA12, bla*_NDM-5_ *, sul1, tet(B), mph(A), bla*_CTX-M-15_
	DL-069-SS1	Solid waste		361		*bla*_TEM-1B_ *, dfrA12, bla*_NDM-5_ *, sul1, tet(B), mph(A), bla*_CTX-M-15_
3	TR-019-FPW	Pond water	Yes	405	IncIγ/K1	*bla*_CMY-42_
	TR-104-DRW	River water		405		*bla*_CMY-42_
	DL-166-WW2	Wastewater	No	8346		*bla*_CMY-42_
4	DL-187-WW1	Wastewater	Yes	448	IncX4	*bla*_TEM-1B_
	DL-181-WW1	Wastewater		448		*bla*_TEM-1B_
5	DL-182-FP1	Poultry pen	Yes	405	IncFIA	*tet(B), bla*_OXA-1_ *, aac(6')-Ib-cr*
	DL-182-CL	Chicken cloacae		405		*tet(B), bla*_OXA-1_ *, aac(6')-Ib-cr*
	DL-182-SS1	Solid waste		405		*tet(B), bla*_OXA-1_ *, aac(6')-Ib-cr*
6	DL-182-CL	Chicken cloacae	Yes	405	IncHI1B	*mph(A), aac(3)-Iia, bla*_CTX-M-15_ *, dfrA17, aadA5, sul1*
	DL-182-SS1	Solid waste		405		*mph(A), aac(3)-Iia, bla*_CTX-M-15_ *, dfrA17, aadA5, sul1*
7	DL-187-SS1	Solid waste	Yes	405	IncFIA	*bla*_NDM-5_ *, tet(B), bla*_TEM-1B_ *, mph(A), dfrA12, aadA2, sul1*
	DL-183-SS1	Solid waste		405		*bla*_NDM-5_ *, tet(B), bla*_TEM-1*B*_ *, mph(A), dfrA12, aadA2, sul1*

### Carriage of mobile genetic elements

A total of 285 MGEs were identified among the isolates, with 50% of these located in contigs containing plasmid replicon types. The top five MGEs found in the chromosome were ISEc9 (52.1% [61/117]), ISKpn19 (21.4% [25/117]), MITEEc1 (12.0% [14/117]), IS609 (10.3% [12/117]), and IS26 (8.5% [10/117]) (Table 8). Of all the isolates, three isolates contained five or more MGEs. Two of these isolates were from a rural household, one from a human with low poultry exposure (TR-113-01-FL-E2), and the other from a wastewater sample (TR-111-WW-E1). The other isolate was from a poultry vendor in a wet market (DL-182-01-FH-K1). All MGEs found in these three isolates were on contigs predicted to be part of the plasmid (Table 8).

**TABLE 8:** Most common MGEs (up to five listed) predicted either in chromosome or in plasmid

MGE (*n*)	No. (%) of samples		
	Poultry (*n* = 12)	Human (*n* = 20)	Environment (*n* = 85)
Chromosome			
ISEc9 (56)	6 (28.5)	14 (23.3)	41 (68.3)
ISKpn19 (25)	2 (8.0)	9 (36.0)	14 (56.0)
MITEEc1 (14)	2 (14.2)	3 (21.4)	9 (64.3)
IS609 (12)	1 (8.3)	4 (33.3)	7 (58.3)
IS26 (10)	1 (10.0)	2 (20.0)	7 (70.0)
Plasmid			
IS6100 (32)	4 (12.5)	5 (15.6)	23 (71.9)
ISKpn19 (13)	3 (23.1)	1 (7.7)	9 (69.2)
ISEc9 (13)	1 (7.7)	2 (15.4)	10 (76.9)
ISSen9 (6)	0	1 (16.7)	5 (83.3)
IS26 (6)	1 (16.7)	0	5 (83.3)

### Characterization of AMR plasmids by long-read sequencing

**Fig 4:**
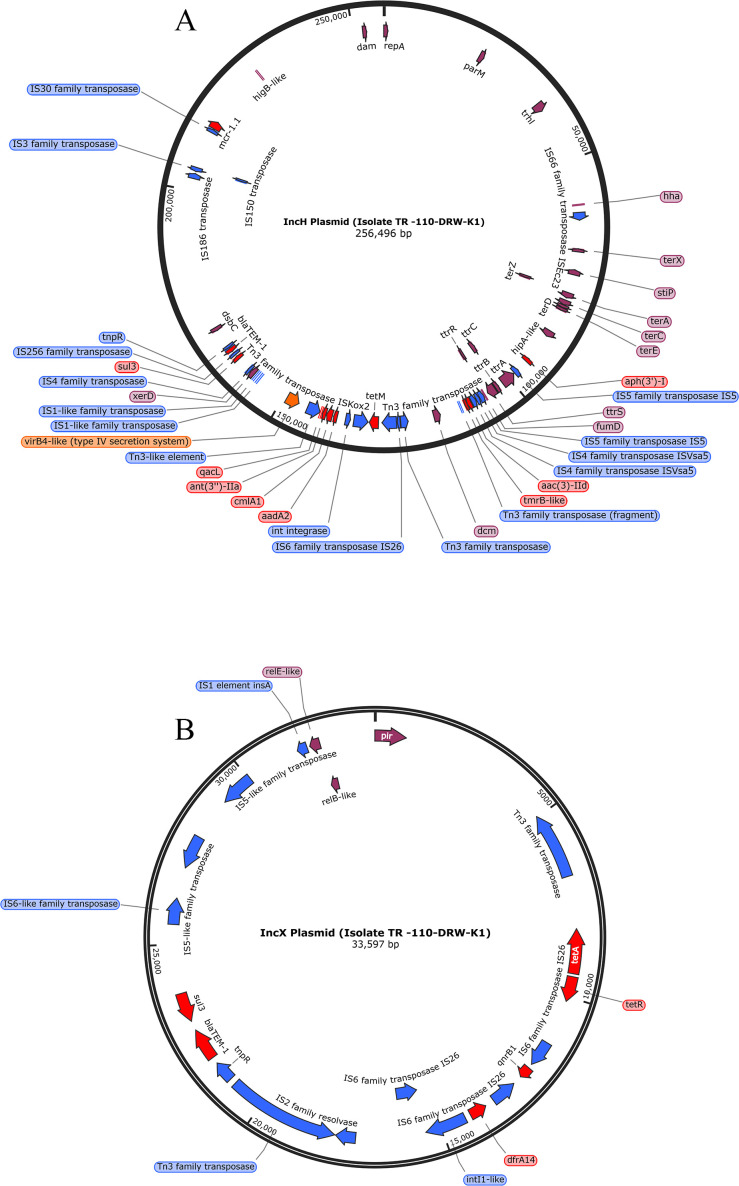
Partial maps of (**A**) IncH plasmid (256,496 bp, fully circularized) and (**B**) IncX plasmid (33,597 bp, fully circularized) recovered from isolate TR-110-DRW-K1. Annotations based on PROKKA output. Genes encoding hypothetical proteins are not shown. Additional curation of annotations was performed with CARD protein BLAST (for antibiotic resistance genes) and UniProt BLAST. Only ARGs with identity and coverage ≥90% are shown. Blue annotations denote potential mobile genetic elements, red annotations indicate antibiotic/biocide resistance genes, orange annotations correspond to virulence genes, and purple annotations relate to all other annotations.

**Fig 5:**
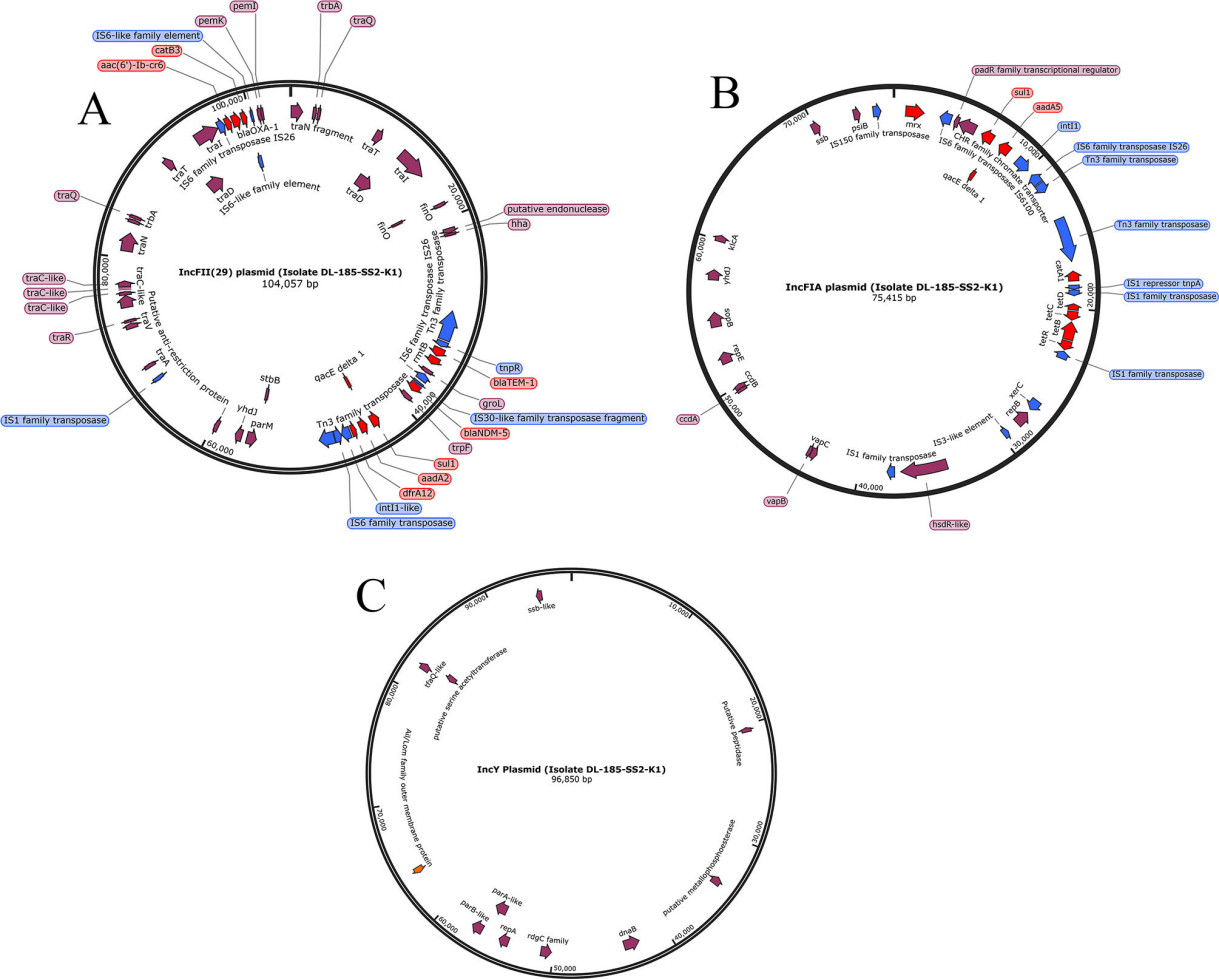
Partial maps of (**A**) IncFII plasmid (104,057 bp, un-circularized), (**B**) IncFIA plasmid (75,415 bp, un-circularized), and (**C**) IncY plasmid (95,850 bp, fully circularized) recovered from isolate DL-185-SS2-K1. Circular maps of un-circularized plasmids are shown for visualization purposes. Annotations based on PROKKA output. Genes encoding hypothetical proteins are not shown. Additional curation of annotations was performed with CARD protein BLAST (for antibiotic resistance genes) and UniProt BLAST. Only ARGs with identity and coverage ≥90% are shown. Blue annotations denote potential mobile genetic elements, red annotations indicate antibiotic/biocide resistance genes, orange annotations correspond to virulence genes, and purple annotations relate to all other annotations.

To further characterize the plasmids, we performed long-read sequencing of two ESBL-producing and carbapenem-resistant isolates, TR-110-DRW-K1 and DL-185-SS2-K1, representing rural and urban areas, respectively. TR-110-DRW-K1 was a CR-Ec isolated from a river water sample in a rural area. The Trycycler consensus assembly of the long-read sequences fully circularized two plasmids. One of these was a large plasmid (~256,496 bp) of IncH type that carried several AMR genes, including *bla*_TEM-1_ associated with resistance to β-lactam/s antibiotics, as well as the *mcr 1.1* determinant for colistin resistance. The IncH plasmid shared considerable sequence homology with a 257,270 bp IncHI2 plasmid pRS571 [CPO34390.1], which was previously identified in *E. coli* isolated from a rectal swab sample taken from a healthy human in Dhaka, Bangladesh (NCBI blast: identity 99.97%, coverage 90%). Other AMR genes in the plasmid were associated with tetracycline, aminoglycosides, sulfonamide, phenicol, and biocide resistance (for specific genes, see Table S5) (Fig. 4A). The other plasmid was an IncX type with an estimated size of 33,597 bp, which carried the *bla*_TEM-1_, β-lactam resistance gene, in addition to resistance determinants for tetracycline, sulfonamide, and trimethoprim resistance (Fig. 4B). Three plasmids were partially assembled from long-read sequences of isolate DL-185-SS2-K1; the largest one was the IncFII type with an estimated size of 104,057 bp, followed by IncY (96,850 bp) and IncFIA (75,415 bp). Among these three plasmids, the IncFII type carried multiple AMR genes, including genetic determinants for β-lactams (*bla*_OXA-1_, *bla*_TEM-1_) and carbapenems (*bla*_NDM-5_). Other genes were associated with phenicol, aminoglycoside, sulfonamide, trimethoprim, and biocide resistance (Fig. 5A). The IncFIA plasmid also carried genes conferring resistance to phenicol, aminoglycoside, tetracycline, and biocides (Fig. 5B). The fully circularized IncY plasmid did not carry any characterized AMR genes (Fig. 5C).

## DISCUSSION

This study aimed to understand AMR transmission between poultry and humans, encompassing direct and indirect exposures. To accomplish this, we analyzed the WGS of 117 ESBL-Ec and CR-Ec isolates obtained from poultry, humans, and the environments that were temporally and spatially connected from three ecological sites. ARG diversity was similar in human isolates regardless of poultry exposure. In contrast, environmental isolates showed higher diversity, especially in aminoglycoside and carbapenem resistance genes, compared to human and poultry isolates, while tetracycline and trimethoprim resistance genes were more prevalent in poultry isolates. Nevertheless, certain ARGs, including those that are associated with colistin, rifampicin, and fosfomycin resistance, were only detected in wastewater isolates. Variations in ARG diversity were also observed among isolates from different wastewater sources. Isolates from wet market wastewater had greater ARG diversity compared to the isolates from household/poultry farm wastewater. The unique environments of urban wet markets contribute to the observed greater ARG diversity in market wastewater. For example, wet markets, which sell a range of products, including livestock, poultry, and fresh produce, generate wastewater with diverse contaminants. Onsite poultry slaughtering also takes place in wet markets. In contrast, rural farm wastewater is predominantly influenced by poultry feces and farm waste.

Because all the selected isolates for this study were ESBL producers, including the carbapenem-resistant ones, we further investigated the distribution of β-lactamase gene subtypes. Interestingly, we found a higher diversity of β-lactamase genes in poultry isolates, a relationship that has not been extensively studied. Three main subtypes (*bla*_CTX-M-15_, *bla*_TEM-1b_, and *bla*_OXA-1_) were prevalent across all sources, with human isolates predominantly positive for *bla*_CTX-M-15_. Moreover, *bla*_CTX-M-15_ in human isolates was primarily detected in contigs linked to chromosomes, indicating clonal expansion as a primary mode of transmission of *bla*_CTX-M-15_ genes among human isolates. The presence of *bla*_CTX-M-15_ in the chromosome, as demonstrated by long-read sequencing of the isolate TR-110-DRW-K1, further attests the clonal expansion mechanism. In the case of *bla*_TEM_ genes, a higher diversity of subtypes was observed in poultry isolates, while human isolates were only positive for *bla*_TEM-1b_. In contrast to *bla*_CTX-M-15_, *bla*_TEM-1b_ was more frequently found in the plasmid contigs of all poultry isolates and most human and environmental isolates, indicating potential transmission of *bla*_TEM-1b_ between human and poultry isolates through plasmid transfer. However, given the lack of evidence of plasmid transfer between human and poultry isolates in this study, other mobile genetic elements might be involved in the transmission of ARGs, which requires further investigation.

Analysis of MGEs among the isolates revealed that ISEc9, carrying *bla*_CTX-M-15_, was primarily located in the chromosome, aligning with the results of a prior study (55). The presence of the *bla*_CTX-M-15_-carrying mobile element in the chromosome suggests that selective pressure may not be a factor in these isolates retaining the resistance gene. To investigate further, we analyzed the clonal diversity of isolates and characterized plasmids carrying ARGs. We found that human isolates exhibited greater genomic diversity than poultry isolates, which may be attributed to human exposure to a wide range of outdoor environments compared to poultry raised in a confined system (56, 57). A previous study in Ghana also reported a higher diversity of ST among human ESBL-Ec isolates than poultry isolates (58). We found that approximately 42% of human and poultry isolates had matched STs with environmental isolates, indicating that the environment is frequently contaminated with ESBL-Ec from both human and animal sources. ST405 was the most common ST observed across all three sources, which is an emerging uropathogenic *E. coli* known to frequently carry ESBL and carbapenem resistance genes and a repertoire of virulence genes similar to another critical group of uropathogenic *E. coli* ST131 (59).

By using core genome SNP analysis, we identified two sequence types (ST155 and ST206) in human isolates that were also found in poultry isolates. However, they were not clonally related (>22 SNPs). Hence, in this study, we did not detect any clonal overlaps between poultry and human isolates, indicating that occupational exposure to poultry may not result in colonization with poultry-adapted ESBL-Ec isolates. However, findings from this study are constrained by the small sample size and analytical approaches. Similar to findings in Vietnam, non-intensive chicken farming showed minimal impact on human ESBL-Ec colonization (60). Nevertheless, we cannot dismiss the possibility of indirect transmission through exposure to contaminated farm environments. In our study, we identified several clonal overlaps between human or poultry isolates and environmental isolates occurring within rural and urban sites. Specifically, clonal overlaps were more prevalent between human and environmental isolates (*n* = 5) than poultry and environmental isolates (*n* = 2). This observation of clonal overlaps being more prevalent between human and environmental isolates contrasts with findings from a prior study, which demonstrated a higher prevalence of shared bacterial genera between wastewater and animals than between wastewater and humans (61). In our study, the poultry isolates mainly originated from broilers raised in confined environments and had minimal exposure to outdoor environments. Farm waste was directly discharged into the environment, which humans frequently accessed. Consequently, transmission between broilers and the environment was more likely to be unidirectional (from broilers to the environment), while for humans, it could occur in both directions.

Although the environment is a significant reservoir for ESBL-Ec originating from diverse sources, surprisingly, we found no clonal overlaps between human and poultry-adapted ESBL-Ec derived from environmental samples. Our prior study on *E. coli* isolates from humans, chickens, cattle, and soil sources in rural households in Bangladesh further substantiates this finding, revealing no clonal overlaps between chicken and human isolates (62). Furthermore, compared to clinical isolates from other countries, the isolates analyzed in this study displayed a degree of heterogeneity. Most of the clinical isolates were found to be of ST131 lineage. In this study, two isolates were identified as ST131 (O25:H4): one was obtained from an individual with higher exposure to poultry, and another was collected from a river water sample downstream of a rural household. Both isolates were clonal (<22 SNP difference) to an ST131 clinical strain (SRR11496578) reported in the USA in 2012 (63). Although there is no discernible epidemiological connection between these isolates, it is crucial to emphasize that ST131 strains are pandemic and have been linked to extraintestinal infections in humans globally (64). Investigating the pathogenic potential of ST131 isolates obtained from non-clinical sources would be interesting. The diversity of ESBL-Ec in our study corresponds with findings from similar studies conducted in various countries. A recent study in Kenya employing WGS on numerous *E. coli* isolates revealed that the diversity and transmission of *E. coli* between humans and animals were significantly influenced by the type of host (11). However, additional research is crucial to investigate the factors governing the selection of host-adapted *E. coli* or the host microbiota that regulate the colonization with specific *E. coli*.

The presence of a plasmid significantly impacts the resistome composition, as isolates harboring a plasmid tended to possess a greater number of resistance genes (Fig. S3). In this study, we did not find a correlation between the presence and type of plasmids and the source of the isolates (human, environment, poultry). We delved into whether plasmid sharing, rather than clonal expansion, was a prevalent mode of AMR transmission in these environments. Our findings indicated no instances of plasmid sharing between human and poultry isolates. Instead, the majority of isolates sharing similar plasmids were obtained from environmental sources, specifically from wastewater and solid waste samples in urban markets. Moreover, in six out of seven instances, isolates sharing a plasmid were also clonally related, suggesting that the dissemination of AMR genes was more likely due to the transfer of the strain alongside the plasmid as opposed to independent plasmid transfer (60). To get more information on the carriage of AMR genes by the plasmids, we performed long-read sequencing of two isolates; both were carbapenem resistant. TR-110-DRW-K1, obtained from a river water sample in a rural area, belonged to ST8346 and serotype O64:H10 and was clonally related to *E. coli* DL-083-SS2, obtained from a solid waste sample in an urban wet market in Dhaka. Two plasmids, IncH and IncX, were circularized from this strain, both carried multiple AMR genes. Interestingly, none of these plasmids carried carbapenem resistance genes, although the isolate was phenotypically resistant to carbapenem and was positive for *bla*_NDM-5_ according to short-read sequencing data (16). In contrast, this isolate was sensitive to colistin despite the presence of a complete colistin resistance gene *mcr1.1* in the IncH plasmid (16). The other isolate (DL-185-SS2-K1) subjected to long-read sequencing was obtained from a solid waste sample from the wet market. The isolate was resistant to all antibiotics tested in the study except colistin and was clonally related to DL-182–02-FHK1, a carbapenem-resistant isolate obtained from human feces with high exposure to poultry environments in the wet market. Of the three plasmids detected in this isolate, IncFII carried multiple AMR genes, including a metallo-β-lactamase gene conferring resistance to carbapenem. Interestingly, for both isolates, we did not find evidence of plasmid sharing with the clonally related strains (100% core genome ANI). Further studies to evaluate the functionality of the AMR genes carried by these plasmids, as well as their transferability to other bacterial species, will help comprehend the impact of these plasmids on the transmission of AMR.

Although our research offers significant insights, some notable limitations include the cross-sectional sampling and a relatively small sample size, which might have impacted the identification of clonal overlaps. Additionally, we analyzed one isolate per sample for WGS, a choice that may not fully capture the overall genetic diversity. The identification of AMR plasmid sharing between isolates was based on partially reconstructed plasmid sequences using contigs from short-read sequencing data and detecting AMR genes in those constructs. Short-read sequencing data do not enable accurate assembly of highly repetitive or homologous regions of the genome. This limitation can lead to incomplete or fragmented assembly of plasmids and chromosomes, potentially affecting the accuracy of prediction of plasmids using the available bioinformatic tools, including MOB-suite (65). Long-read sequencing technologies can span these complex regions more effectively, leading to more accurate and contiguous assemblies. Furthermore, due to the absence of appropriate biosecurity measures, including rodent and pest control in the poultry farms we investigated, it is conceivable that ESBL-Ec originating from various sources, such as insects or rodents, could have been transmitted to the farm environment and subsequently to poultry (60, 66–69). However, this aspect was not explored in our study.

### Conclusion

In conclusion, our study provides insights into the complexity of AMR transmission between poultry, humans, and the environment in Bangladesh. We found no significant difference in the diversity of ARGs between isolates from individuals with high and low exposure to poultry. Environmental isolates exhibited higher ARG diversity than human and poultry isolates. Although a clonal overlap between poultry and human isolates was not detected, it was evident that the environment possessed ESBL-Ec from both poultry and human sources. The presence of ESBL-Ec in the environment is probably attributed to the discharge of human and animal wastes into the environment. However, exposure to contaminated environments may not always result in the colonization of humans with poultry-adapted ESBL-Ec, and vice versa. The sharing of strains with plasmids was more likely linked with the spread of AMR genes than independent plasmid transfer. Overall, our study highlights the necessity for a system mapping for AMR transmission, enabling the identification of targeted interventions to address this multifaceted problem effectively.

## Data Availability

Genomic data are available from the European Nucleotide Archive; study accession PRJEB48068. Other data relating to the wider project are openly accessible at https://doi.org/10.5285/0239cdaf-deab-4151-8f68-715063eaea45 and https://doi.org/10.5285/dda6dd55-f955-4dd5-bc03-b07cc8548a3d.

## SUPPLEMENTAL MATERIAL

Fig. S1 spectrum.03956-23-s0001.tiff Presence of resistance and virulence genes with plasmid types.

Fig. S2 spectrum.03956-23-s0002.tiff Violin plot comparing the ARG diversity among wastewater isolates.

Fig. S3 spectrum.03956-23-s0003.tiff Violin plot comparing the total resistance genes between samples without plasmids (teal) and those with plasmids (orange).

Fig. S4 spectrum.03956-23-s0004.tiff Grapetree displaying an MSTree v2 generated using cgMLST V1 + HierCC V1.

Supplemental material spectrum.03956-23-s0005.docx Table S1 to S5; figure legends.

ASM does not own the copyrights to Supplemental Material that may be linked to, or accessed through, an article. The authors have granted ASM a non-exclusive, world-wide license to publish the Supplemental Material files. Please contact the corresponding author directly for reuse.

## References

[1] Mendelson M, Matsoso MP. 2015. The world health organization global action plan for antimicrobial resistance. S Afr Med J 105:325. doi:10.7196/samj.9644 26242647

[2] Laxminarayan R, Chaudhury RR. 2016. Antibiotic resistance in India: drivers and opportunities for action. PLoS Med 13:e1001974. doi:10.1371/journal.pmed.1001974 26934098 PMC4775002

[3] Rousham E, Unicomb L, Wood P, Smith M, Asaduzzaman M, Islam MA. 2018. Spatial and temporal variation in the community prevalence of antibiotic resistance in Bangladesh: an integrated surveillance study protocol. BMJ Open 8:e023158. doi:10.1136/bmjopen-2018-023158 PMC5931287 29705771

[4] Herrero M, Grace D, Njuki J, Johnson N, Enahoro D, Silvestri S, Rufino MC. 2013. The roles of livestock in developing countries. Animal 7 Suppl 1:3–18. doi:10.1017/S1751731112001954 23121696

[5] Devendra C, Chantalakhana C. 2002. Animals, poor people and food insecurity: opportunities for improved livelihoods through efficient natural resource management. Outlook Agric 31:161–175. doi:10.5367/000000002101294010

[6] Thornton PK. 2010. Livestock production: recent trends, future prospects. Philos Trans R Soc Lond B Biol Sci 365:2853–2867. doi:10.1098/rstb.2010.0134 20713389 PMC2935116

[7] Alam M-U, Rahman M, Abdullah-Al M, Islam MA, Asaduzzaman M, Sarker S, Rousham E, Unicomb L. 2019. Human exposure to antimicrobial resistance from poultry production: assessing hygiene and waste-disposal practices in Bangladesh. Int J Hyg Environ Health 222:1068–1076. doi:10.1016/j.ijheh.2019.07.007 31331788

[8] Rimi NA, Sultana R, Muhsina M, Uddin B, Haider N, Nahar N, Zeidner N, Sturm-Ramirez K, Luby SP. 2017. Biosecurity conditions in small commercial chicken farms, Bangladesh 2011-2012. Ecohealth 14:244–258. doi:10.1007/s10393-017-1224-2 28289988 PMC5942227

[9] Masud AA, Rousham EK, Islam MA, Alam MU, Rahman M, Mamun AA, Sarker S, Asaduzzaman M, Unicomb L. 2020. Drivers of antibiotic use in poultry production in Bangladesh: dependencies and dynamics of a patron-client relationship. Front Vet Sci 7:78. doi:10.3389/fvets.2020.00078 32185184 PMC7058630

[10] Rousham EK, Asaduzzaman M, Mozmader TIMAU, Amin MB, Rahman M, Hossain MI, Islam MR, Mahmud ZH, Unicomb L, Islam MA. 2021. Human Colonization with extended-spectrum beta-Lactamase-producing E. coli in relation to animal and environmental exposures in Bangladesh: An observational one health study. Environ Health Perspect 129:37001. doi:10.1289/EHP7670 33656920 PMC7929562

[11] Muloi DM, Wee BA, McClean DMH, Ward MJ, Pankhurst L, Phan H, Ivens AC, Kivali V, Kiyong’a A, Ndinda C, et al.. 2022. Population genomics of Escherichia coli in livestock-keeping households across a rapidly developing urban landscape. Nat Microbiol 7:581–589. doi:10.1038/s41564-022-01079-y 35288654 PMC8975746

[12] Rousham EK, Unicomb L, Islam MA. 2018. Human, animal and environmental contributors to antibiotic resistance in low-resource settings: integrating behavioural, epidemiological and one health approaches. Proc Biol Sci 285:20180332. doi:10.1098/rspb.2018.0332 29643217 PMC5904322

[13] WHO. 2017. WHO publishes list of bacteria for which new antibiotics are urgently needed. Available from: http://www.who.int/mediacentre/news/releases/2017/bacteria-antibiotics-needed/en/

[14] Tang HJ, Hsieh CF, Chang PC, Chen JJ, Lin YH, Lai CC, Chao CM, Chuang YC. 2016. Clinical significance of community- and healthcare-acquired carbapenem-resistant Enterobacteriaceae isolates. PLoS One 11:e0151897. doi:10.1371/journal.pone.0151897 26999356 PMC4801408

[15] Centers for Disease Control and Prevention. 2019. Centers for disease control and prevention: antibiotic resistance threats in the United States, 2019. Atlanta, GA Centers for Disease Control and Prevention

[16] Asaduzzaman M, Rousham E, Unicomb L, Islam MR, Amin MB, Rahman M, Hossain MI, Mahmud ZH, Szegner M, Wood P, Islam MA. 2022. Spatiotemporal distribution of antimicrobial resistant organisms in different water environments in urban and rural settings of Bangladesh. Sci Total Environ 831:154890. doi:10.1016/j.scitotenv.2022.154890 35364179

[17] Amin MB, Hoque KI, Roy S, Saha SR, Islam M, Julian TR, Islam MA. 2022. Identifying the sources of intestinal colonization with extended-spectrum beta-lactamase-producing Escherichia coli in healthy infants in the community. Front Microbiol 13:803043. doi:10.3389/fmicb.2022.803043 35432268 PMC9008759

[18] Andrews S. 2010. Fastqc: a quality control tool for high throughput sequence data

[19] Clausen P, Aarestrup FM, Lund O. 2018. Rapid and precise alignment of raw reads against redundant databases with KMA. BMC Bioinformatics 19:307. doi:10.1186/s12859-018-2336-6 30157759 PMC6116485

[20] Joensen KG, Scheutz F, Lund O, Hasman H, Kaas RS, Nielsen EM, Aarestrup FM. 2014. Real-time whole-genome sequencing for routine typing, surveillance, and outbreak detection of verotoxigenic Escherichia coli. J Clin Microbiol 52:1501–1510. doi:10.1128/JCM.03617-13 24574290 PMC3993690

[21] Malberg Tetzschner AM, Johnson JR, Johnston BD, Lund O, Scheutz F. 2020. In silico genotyping of Escherichia coli isolates for extraintestinal virulence genes by use of whole-genome sequencing data. J Clin Microbiol 58:e01269-20. doi:10.1128/JCM.01269-20 32669379 PMC7512150

[22] Bortolaia V, Kaas RS, Ruppe E, Roberts MC, Schwarz S, Cattoir V, Philippon A, Allesoe RL, Rebelo AR, Florensa AF, et al.. 2020. Resfinder 4.0 for predictions of phenotypes from genotypes. J Antimicrob Chemother 75:3491–3500. doi:10.1093/jac/dkaa345 32780112 PMC7662176

[23] Zankari E, Allesøe R, Joensen KG, Cavaco LM, Lund O, Aarestrup FM. 2017. Pointfinder: a novel web tool for WGS-based detection of antimicrobial resistance associated with chromosomal point mutations in bacterial pathogens. J Antimicrob Chemother 72:2764–2768. doi:10.1093/jac/dkx217 29091202 PMC5890747

[24] Carattoli A, Zankari E, García-Fernández A, Voldby Larsen M, Lund O, Villa L, Møller Aarestrup F, Hasman H. 2014. In silico detection and typing of plasmids using plasmidfinder and plasmid multilocus sequence typing. Antimicrob Agents Chemother 58:3895–3903. doi:10.1128/AAC.02412-14 24777092 PMC4068535

[25] Beghain J, Bridier-Nahmias A, Le Nagard H, Denamur E, Clermont O. 2018. Clermontyping: an easy-to-use and accurate in silico method for Escherichia genus strain phylotyping. Microb Genom 4:e000192. doi:10.1099/mgen.0.000192 29916797 PMC6113867

[26] Zhou Z, Alikhan NF, Mohamed K, Fan Y, Achtman M, Agama Study Group. 2020. The Enterobase user’s guide, with case studies on Salmonella transmissions, Yersinia pestis phylogeny, and Escherichia core genomic diversity. Genome Res 30:138–152. doi:10.1101/gr.251678.119 31809257 PMC6961584

[27] Gu Z, Eils R, Schlesner M. 2016. Complex heatmaps reveal patterns and correlations in multidimensional genomic data. Bioinformatics 32:2847–2849. doi:10.1093/bioinformatics/btw313 27207943

[28] Durrant MG, Li MM, Siranosian BA, Montgomery SB, Bhatt AS. 2020. A bioinformatic analysis of integrative mobile genetic elements highlights their role in bacterial adaptation. Cell Host Microbe 27:140–153. doi:10.1016/j.chom.2019.10.022 31862382 PMC6952549

[29] Fisher RA. 1925. Statistical methods for research workers. Oliver and Boyd, Edinburgh, London.

[30] Patil I. 2021. Visualizations with statistical details: the 'ggstatsplot' approach. JOSS 6:3167. doi:10.21105/joss.03167

[31] Oksanen JS, Blanchet F, Kindt R, Legendre P, Minchin P, O’Hara R R, Stevens M, Szoecs E, Wagner H, Barbour M, Bedward M, Borcard D, Carvalho G, Chirico M, De Caceres MD, Evangelista H, FitzJohn R, Friendly M, Furneaux B. 2022. _Vegan: Community Ecology package_. R package version 2.6-4

[32] Wick RR, Judd LM, Gorrie CL, Holt KE. 2017. Unicycler: resolving bacterial genome assemblies from short and long sequencing reads. PLoS Comput Biol 13:e1005595. doi:10.1371/journal.pcbi.1005595 28594827 PMC5481147

[33] Schwengers O, Jelonek L, Dieckmann MA, Beyvers S, Blom J, Goesmann A. 2021. Bakta: rapid and standardized annotation of bacterial genomes via alignment-free sequence identification. Microbial Genomics 7:000685. doi:10.1099/mgen.0.000685 34739369 PMC8743544

[34] Page AJ, Cummins CA, Hunt M, Wong VK, Reuter S, Holden MTG, Fookes M, Falush D, Keane JA, Parkhill J. 2015. Roary: rapid large-scale prokaryote pan genome analysis. Bioinformatics 31:3691–3693. doi:10.1093/bioinformatics/btv421 26198102 PMC4817141

[35] Page AJ, Taylor B, Delaney AJ, Soares J, Seemann T, Keane JA, Harris SR. 2016. SNP-sites: rapid efficient extraction of SNPs from multi-FASTA alignments. Microb Genom 2:e000056. doi:10.1099/mgen.0.000056 28348851 PMC5320690

[36] Nguyen L-T, Schmidt HA, von Haeseler A, Minh BQ. 2015. IQ-TREE: a fast and effective stochastic algorithm for estimating maximum-likelihood phylogenies. Mol Biol Evol 32:268–274. doi:10.1093/molbev/msu300 25371430 PMC4271533

[37] Letunic I, Bork P. 2021. Interactive tree of life (iTOL) v5: an online tool for phylogenetic tree display and annotation. Nucleic Acids Res 49:W293–W296. doi:10.1093/nar/gkab301 33885785 PMC8265157

[38] David S, Reuter S, Harris SR, Glasner C, Feltwell T, Argimon S, Abudahab K, Goater R, Giani T, Errico G, Aspbury M, Sjunnebo S, Feil EJ, Rossolini GM, Aanensen DM, Grundmann H, EuSCAPE Working Group, ESGEM Study Group. 2019. Epidemic of carbapenem-resistant Klebsiella pneumoniae in Europe is driven by nosocomial spread. Nat Microbiol 4:1919–1929. doi:10.1038/s41564-019-0492-8 31358985 PMC7244338

[39] Zhou Z, Alikhan N-F, Sergeant MJ, Luhmann N, Vaz C, Francisco AP, Carriço JA, Achtman M. 2018. GrapeTree: visualization of core genomic relationships among 100,000 bacterial pathogens. Genome Res 28:1395–1404. doi:10.1101/gr.232397.117 30049790 PMC6120633

[40] Robertson J, Bessonov K, Schonfeld J, Nash JHE. 2020. Universal whole-sequence-based plasmid typing and its utility to prediction of host range and epidemiological surveillance. Microb Genom 6:mgen000435. doi:10.1099/mgen.0.000435 32969786 PMC7660255

[41] Robertson J, Nash JHE. 2018. MOB-suite: software tools for clustering, reconstruction and typing of pasmids from draft assemblies. Microb Genom 4:e000206. doi:10.1099/mgen.0.000206 30052170 PMC6159552

[42] Seemann T. 2020 Abricate. Github. https://github.com/tseemann/abricate.

[43] Fernández-de-Bobadilla MD, Talavera-Rodríguez A, Chacón L, Baquero F, Coque TM, Lanza VF. 2021. PATO: pangenome analysis toolkit. Bioinformatics 37:4564–4566. doi:10.1093/bioinformatics/btab697 34623430

[44] Treangen TJ, Ondov BD, Koren S, Phillippy AM. 2014. The harvest suite for rapid core-genome alignment and visualization of thousands of intraspecific microbial genomes. Genome Biol 15:524. doi:10.1186/s13059-014-0524-x 25410596 PMC4262987

[45] Wick RR, Holt KE. 2019. Benchmarking of long-read assemblers for prokaryote whole genome sequencing. F1000Res 8:2138. doi:10.12688/f1000research.21782.4 31984131 PMC6966772

[46] Wences AH, Schatz MC. 2015. Metassembler: merging and optimizing de novo genome assemblies. Genome Biol 16:207. doi:10.1186/s13059-015-0764-4 26403281 PMC4581417

[47] Wick RR, Judd LM, Cerdeira LT, Hawkey J, Méric G, Vezina B, Wyres KL, Holt KE. 2021. Trycycler: consensus long-read assemblies for bacterial genomes. Genome Biol 22:266. doi:10.1186/s13059-021-02483-z 34521459 PMC8442456

[48] Koren S, Walenz BP, Berlin K, Miller JR, Bergman NH, Phillippy AM. 2017. Canu: scalable and accurate long-read assembly via adaptive K-MER weighting and repeat separation. Genome Res 27:722–736. doi:10.1101/gr.215087.116 28298431 PMC5411767

[49] Kolmogorov M, Yuan J, Lin Y, Pevzner PA. 2019. Assembly of long, error-prone reads using repeat graphs. Nat Biotechnol 37:540–546. doi:10.1038/s41587-019-0072-8 30936562

[50] Ruan J, Li H. 2020. Fast and accurate long-read assembly with wtdbg2. Nat Methods 17:155–158. doi:10.1038/s41592-019-0669-3 31819265 PMC7004874

[51] Vaser R, Šikić M. 2021. Time-and memory-efficient genome assembly with raven. Nat Comput Sci 1:332–336. doi:10.1038/s43588-021-00073-4 38217213

[52] Wick RR, Judd LM, Gorrie CL, Holt KE. 2017. Unicycler: resolving bacterial genome assemblies from short and long sequencing reads. PLoS Comput Biol 13:e1005595. doi:10.1371/journal.pcbi.1005595 28594827 PMC5481147

[53] Gurevich A, Saveliev V, Vyahhi N, Tesler G. 2013. QUAST: quality assessment tool for genome assemblies. Bioinformatics 29:1072–1075. doi:10.1093/bioinformatics/btt086 23422339 PMC3624806

[54] Seemann T. 2014. Prokka: rapid prokaryotic genome annotation. Bioinformatics 30:2068–2069. doi:10.1093/bioinformatics/btu153 24642063

[55] Grevskott DH, Salvà-Serra F, Moore ERB, Marathe NP. 2020. Nanopore sequencing reveals genomic map of CTX-M-type extended-spectrum beta-lactamases carried by Escherichia coli strains isolated from blue mussels (Mytilus edulis) in Norway. BMC Microbiol 20:134. doi:10.1186/s12866-020-01821-8 32450819 PMC7249450

[56] Ojer-Usoz E, González D, Vitas AI. 2017. Clonal diversity of ESBL-producing Escherichia coli isolated from environmental, human and food samples. Int J Environ Res Public Health 14:676. doi:10.3390/ijerph14070676 28644413 PMC5551114

[57] Mazurek J, Bok E, Baldy-Chudzik K. 2018. Complexity of antibiotic resistance in commensal Escherichia coli derived from pigs from an intensive-production farm. Microbes Environ 33:242–248. doi:10.1264/jsme2.ME17041 30210140 PMC6167118

[58] Falgenhauer L, Imirzalioglu C, Oppong K, Akenten CW, Hogan B, Krumkamp R, Poppert S, Levermann V, Schwengers O, Sarpong N, Owusu-Dabo E, May J, Eibach D. 2018. Detection and characterization of ESBL-producing Escherichia coli from humans and poultry in Ghana. Front Microbiol 9:3358. doi:10.3389/fmicb.2018.03358 30697208 PMC6340976

[59] Roy Chowdhury P, McKinnon J, Liu M, Djordjevic SP. 2018. Multidrug resistant uropathogenic Escherichia coli ST405 with a novel, composite IS26 transposon in a unique chromosomal location. Front Microbiol 9:3212. doi:10.3389/fmicb.2018.03212 30671039 PMC6331395

[60] Nguyen VT, Jamrozy D, Matamoros S, Carrique-Mas JJ, Ho HM, Thai QH, Nguyen TNM, Wagenaar JA, Thwaites G, Parkhill J, Schultsz C, Ngo TH. 2019. Limited contribution of non-intensive chicken farming to ESBL-producing Escherichia coli colonization in humans in Vietnam: an epidemiological and genomic analysis. J Antimicrob Chemother 74:561–570. doi:10.1093/jac/dky506 30629197 PMC6376849

[61] Pal C, Bengtsson-Palme J, Kristiansson E, Larsson DGJ. 2016. The structure and diversity of human, animal and environmental resistomes. Microbiome 4:54. doi:10.1186/s40168-016-0199-5 27717408 PMC5055678

[62] Montealegre MC, Talavera Rodríguez A, Roy S, Hossain MI, Islam MA, Lanza VF, Julian TR. 2020. High genomic diversity and heterogenous origins of pathogenic and antibiotic-resistant Escherichia coli in household settings represent a challenge to reducing transmission in low-income settings. mSphere 5:e00704-19. doi:10.1128/mSphere.00704-19 31941809 PMC6968650

[63] Miles-Jay A, Weissman SJ, Adler AL, Baseman JG, Zerr DM. 2021. Whole genome sequencing detects minimal clustering among Escherichia coli sequence type 131-H30 isolates collected from United States children’s hospitals. J Pediatric Infect Dis Soc 10:183–187. doi:10.1093/jpids/piaa023 32185378 PMC7996643

[64] Pitout JD, DeVinney R. 2017. Escherichia coli St131: a multidrug-resistant clone primed for global domination. F1000Res 6:195. doi:10.12688/f1000research.10609.1 PMC5333602 28344773

[65] Paganini JA, Plantinga NL, Arredondo-Alonso S, Willems RJL, Schürch AC. 2021. Recovering Escherichia coli plasmids in the absence of long-read sequencing data. Microorganisms 9:1613. doi:10.3390/microorganisms9081613 34442692 PMC8400445

[66] Heuer H, Schmitt H, Smalla K. 2011. Antibiotic resistance gene spread due to manure application on agricultural fields. Curr Opin Microbiol 14:236–243. doi:10.1016/j.mib.2011.04.009 21546307

[67] Manyi-Loh C, Mamphweli S, Meyer E, Okoh A. 2018. Antibiotic use in agriculture and its consequential resistance in environmental sources: Potential public health implications. Molecules 23:795. doi:10.3390/molecules23040795 29601469 PMC6017557

[68] Tufa TB, Fuchs A, Wienemann T, Eggers Y, Abdissa S, Schneider M, Jensen B-E, Bode JG, Pfeffer K, Häussinger D, Mackenzie CR, Orth HM, Feldt T. 2020. Carriage of ESBL-producing gram-negative bacteria by flies captured in a hospital and its suburban surroundings in Ethiopia. Antimicrob Resist Infect Control 9:175. doi:10.1186/s13756-020-00836-0 33148323 PMC7640391

[69] Guyomard-Rabenirina S, Reynaud Y, Pot M, Albina E, Couvin D, Ducat C, Gruel G, Ferdinand S, Legreneur P, Le Hello S, Malpote E, Sadikalay S, Talarmin A, Breurec S. 2020. Antimicrobial resistance in wildlife in Guadeloupe (French West Indies): distribution of a single BLA (CTX-M-1)/Inci1/St3 plasmid among humans and wild animals. Front Microbiol 11:1524. doi:10.3389/fmicb.2020.01524 32754130 PMC7366356

