# Supplementary figures and images for: Tracking antimicrobial resistance transmission in urban and rural communities in Bangladesh: a One Health study of genomic diversity of ESBL-producing and carbapenem-resistant Escherichia coli
Source: Microbiol Spectr. 2024 May 3;12(6):e03956-23. doi: 10.1128/spectrum.03956-23 (PMC11237648; doi:10.1128/spectrum.03956-23)

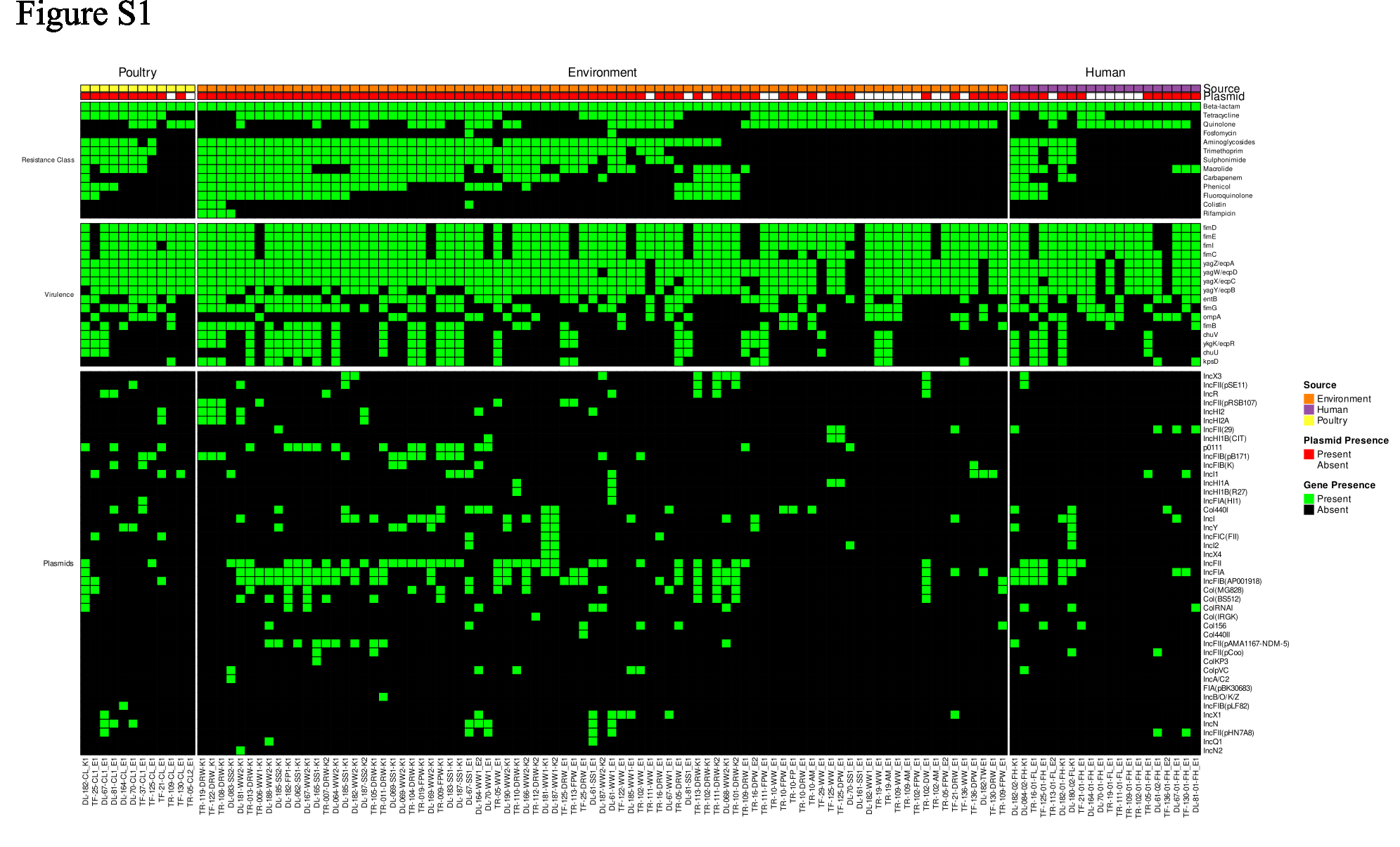

Supplement: Fig. S1 — Presence of resistance and virulence genes with plasmid types. [file spectrum.03956-23-s0001.tiff]

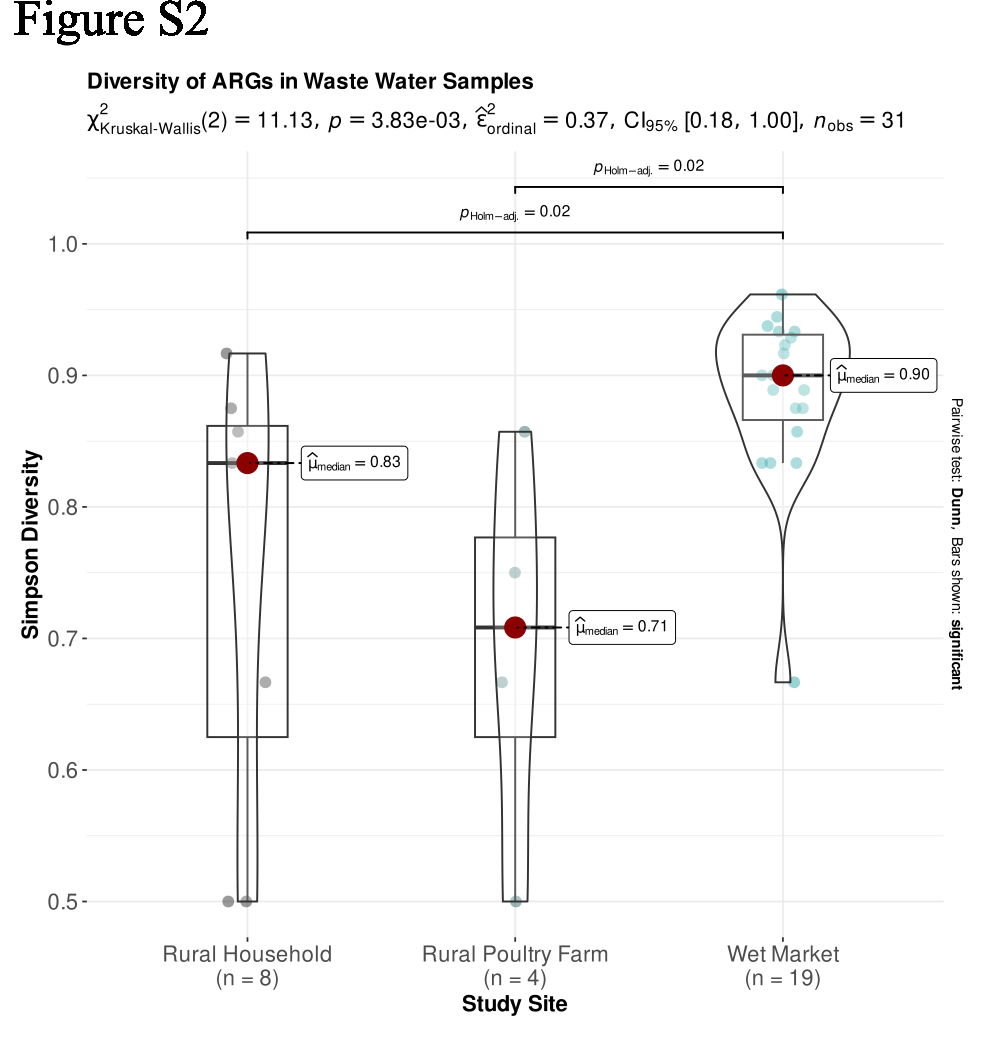

Supplement: Fig. S2 — Violin plot comparing the ARG diversity among wastewater isolates. [file spectrum.03956-23-s0002.tiff]

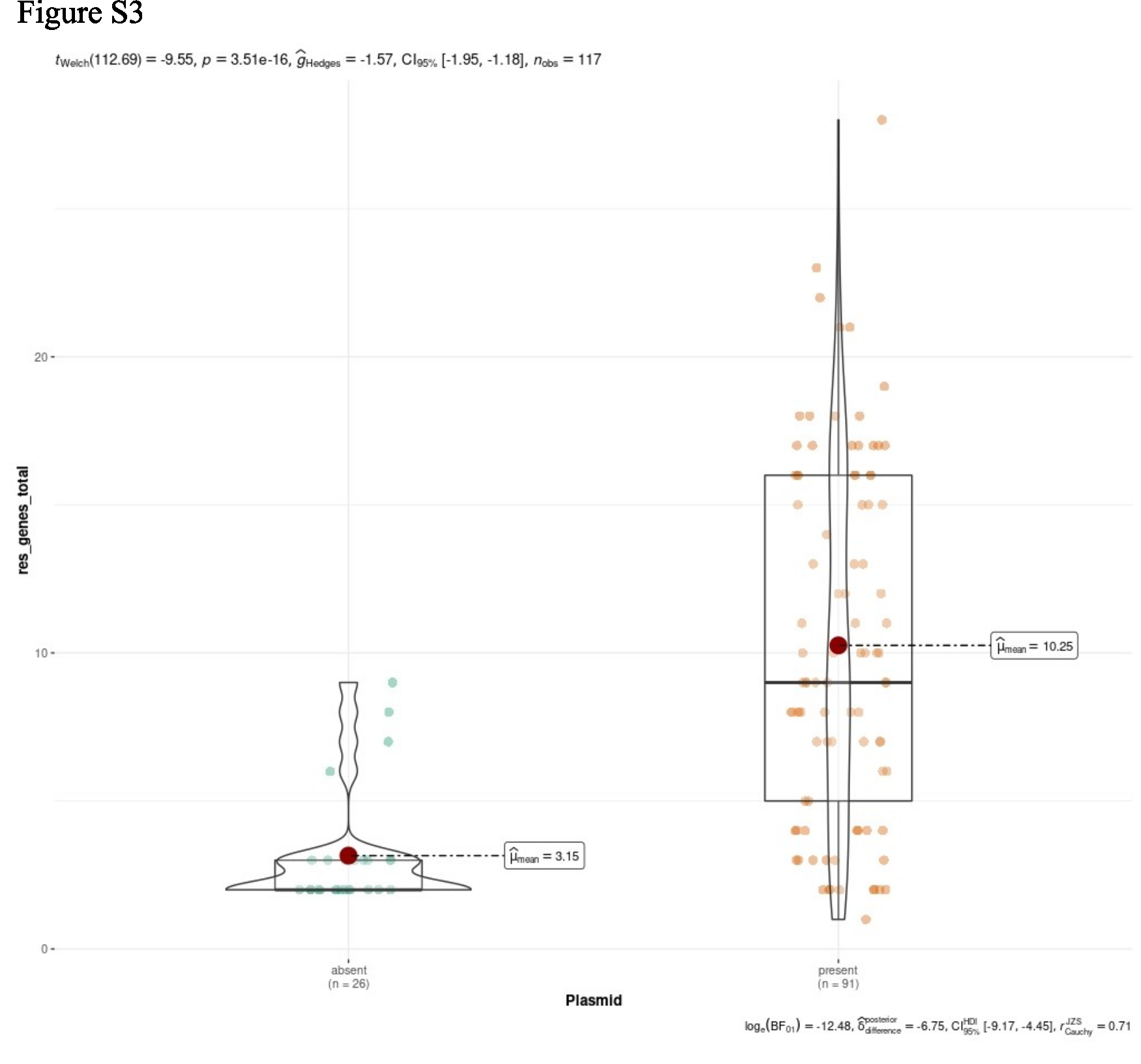

Supplement: Fig. S3 — Violin plot comparing the total resistance genes between samples without plasmids (teal) and those with plasmids (orange). [file spectrum.03956-23-s0003.tiff]

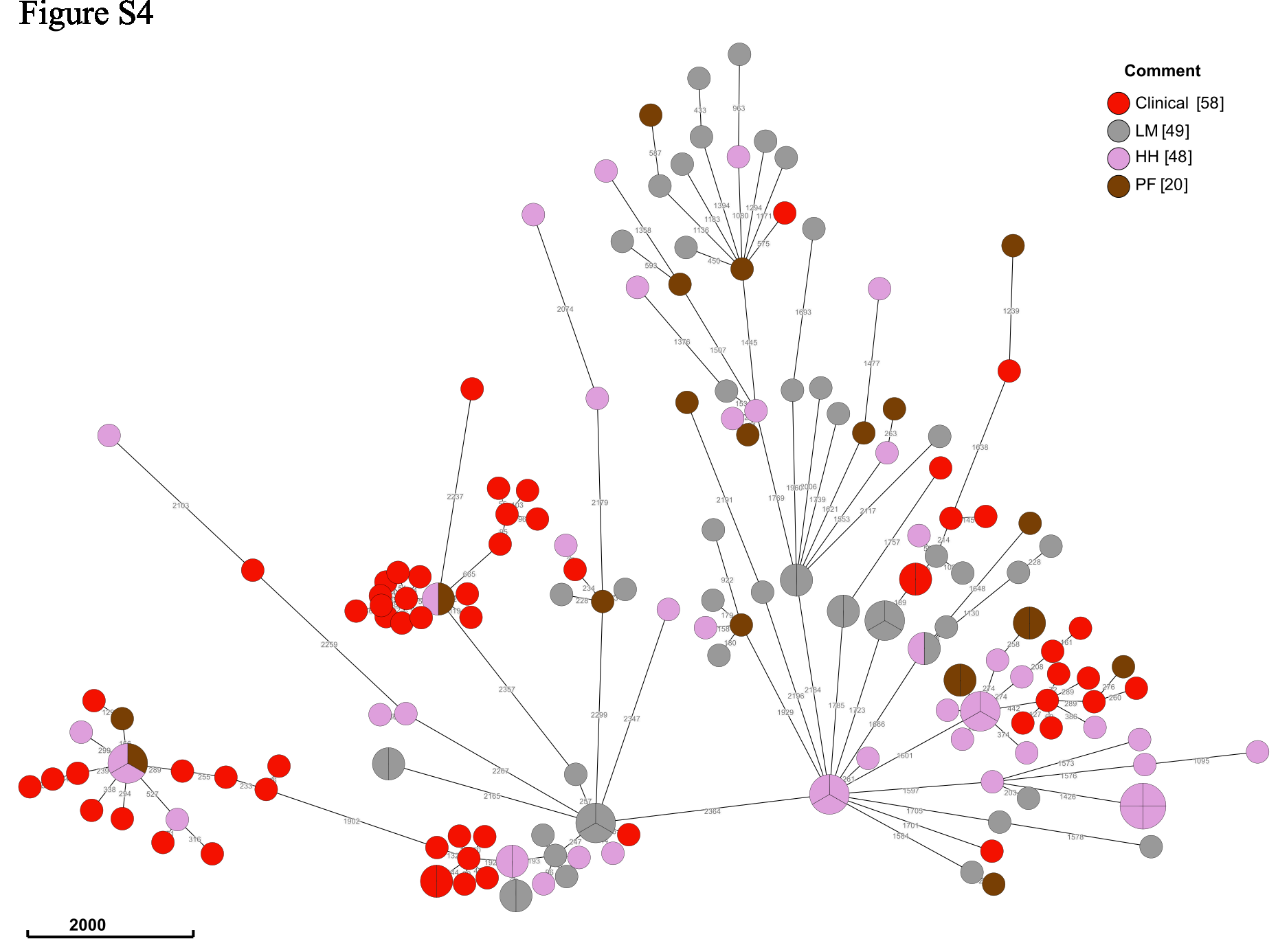

Supplement: Fig. S4 — Grapetree displaying an MSTree v2 generated using cgMLST V1 + HierCC V1. [file spectrum.03956-23-s0004.tiff]
