# Supplementary material for: Tracking antimicrobial resistance transmission in urban and rural communities in Bangladesh: a One Health study of genomic diversity of ESBL-producing and carbapenem-resistant Escherichia coli
Source: Microbiol Spectr. 2024 May 3;12(6):e03956-23. doi: 10.1128/spectrum.03956-23 (PMC11237648; doi:10.1128/spectrum.03956-23)
Supplement: Supplemental material — Table S1 to S5; figure legends. [file spectrum.03956-23-s0005.docx]

**Supplementary files**

**Table S1**. Whole genome sequence data of clinical *E. coli* isolates used for comparative genomic analysis.

| NCBI:BioProject | NCBI:SRP | NCBI:SRS | NCBI:SRX | NCBI:SRR | MLST | Type | Location | Collection Date |
| --- | --- | --- | --- | --- | --- | --- | --- | --- |
| PRJEB17503 | ERP019365 | ERS1434308 | ERX1790609 | ERR1720657 | 131 | urine | Saudi Arabia | 2014 |
| PRJEB17503 | ERP019365 | ERS1434309 | ERX1790610 | ERR1720658 | 448 | urine | Saudi Arabia | 2014 |
| PRJEB17503 | ERP019365 | ERS1443909 | ERX1795918 | ERR1725933 | 448 | urine | Saudi Arabia | 2014 |
| PRJNA600948 | SRP241755 | SRS5985051 | SRX7547499 | SRR10878364 | 38 | blood | Guadeloupe | 2013 |
| PRJNA288601 | SRP074197 | SRS6226151 | SRX7813752 | SRR11193632 | 38 | blood | USA | 2014 |
| PRJNA599369 | SRP253261 | SRS6336662 | SRX7949981 | SRR11347849 | 448 | rectal swab | Qatar:Doha | 2018 |
| PRJNA599369 | SRP253261 | SRS6336474 | SRX7949793 | SRR11348037 | 155 | rectal swab | Qatar:Doha | 2018 |
| PRJNA578285 | SRP255560 | SRS6441426 | SRX8072614 | SRR11496538 | 131 | blood | USA | 2010 |
| PRJNA578285 | SRP255560 | SRS6441422 | SRX8072611 | SRR11496541 | 131 | blood | USA | 2011 |
| PRJNA578285 | SRP255560 | SRS6441397 | SRX8072585 | SRR11496567 | 131 | blood | USA | 2012 |
| PRJNA578285 | SRP255560 | SRS6441386 | SRX8072574 | SRR11496578 | 131 | blood | USA | 2012 |
| PRJNA578285 | SRP255560 | SRS6441372 | SRX8072561 | SRR11496591 | 131 | blood | USA | 2011 |
| PRJNA578285 | SRP255560 | SRS6441284 | SRX8072472 | SRR11496680 | 131 | urine | USA | 2013 |
| PRJNA578285 | SRP255560 | SRS6441264 | SRX8072452 | SRR11496700 | 131 | blood | USA | 2011 |
| PRJNA644880 | SRP270920 | SRS6966405 | SRX8687740 | SRR12172964 | 131 | rectal | Thailand: Songkla | 2017 |
| PRJNA705836 | SRP308781 | SRS8350579 | SRX10204338 | SRR13823711 | 38 | rectal swab | France: Port-Saint-Louis | 2016 |
| PRJNA741123 | SRP325975 | SRS9294423 | SRX11248726 | SRR14936290 | 405 | blood | USA | 2018 |
| PRJNA741123 | SRP325975 | SRS9294419 | SRX11248722 | SRR14936294 | 405 | blood | USA | 2018 |
| PRJNA825705 | SRP369762 | SRS12585857 | SRX14830035 | SRR18729694 | 131 | blood | USA | unreported |
| PRJNA825705 | SRP369762 | SRS12585856 | SRX14830034 | SRR18729695 | 38 | blood | USA | unreported |
| PRJNA825705 | SRP369762 | SRS12585855 | SRX14830033 | SRR18729696 | 448 | blood | USA | unreported |
| PRJNA419720 | SRP125582 | SRS12788336 | SRX15041860 | SRR18965640 | 155 | feces | United Kingdom:Scotland | 2015 |
| PRJNA419720 | SRP125582 | SRS12788664 | SRX15042187 | SRR18965855 | 405 | feces | United Kingdom:Scotland | 2008 |
| PRJNA836696 | SRP374671 | SRS12965192 | SRX15231757 | SRR19165479 | 38 | blood | USA: Houston | 2019 |
| PRJNA836696 | SRP374671 | SRS12965168 | SRX15231733 | SRR19165503 | 38 | blood | USA: Houston | 2018 |
| PRJNA855633 | SRP385048 | SRS13716352 | SRX16036323 | SRR19995253 | 155 | urine | Denmark:Copenhagen | 2019 |
| PRJNA516477 | SRP184283 | SRS4336452 | SRX5343819 | SRR8541306 | 38 | urine | Canada | 2014 |
| PRJNA516477 | SRP184283 | SRS4336263 | SRX5343590 | SRR8541535 | 38 | urine | Canada | 2014 |
| PRJNA522367 | SRP185885 | SRS4363905 | SRX5374854 | SRR8573912 | 131 | feces | France | 2014 |
| PRJNA551371 | SRP212396 | SRS5039759 | SRX6379105 | SRR9615901 | 38 | urine | France | 2017 |
| PRJNA551371 | SRP212396 | SRS5039741 | SRX6379087 | SRR9615919 | 38 | urine | France | 2016 |
| PRJNA551371 | SRP212396 | SRS5039727 | SRX6379073 | SRR9615933 | 38 | urine | France | 2016 |
| PRJNA551371 | SRP212396 | SRS5039717 | SRX6379063 | SRR9615943 | 405 | urine | France | 2016 |
| PRJNA551371 | SRP212396 | SRS5039701 | SRX6379047 | SRR9615959 | 131 | urine | France | 2015 |
| PRJNA551371 | SRP212396 | SRS5039697 | SRX6379044 | SRR9615962 | 405 | urine | France | 2016 |
| PRJNA551371 | SRP212396 | SRS5039658 | SRX6379004 | SRR9616002 | 38 | urine | France | 2016 |
| PRJNA551371 | SRP212396 | SRS5039653 | SRX6378999 | SRR9616007 | 405 | urine | France | 2016 |
| PRJNA551371 | SRP212396 | SRS5039640 | SRX6378986 | SRR9616020 | 405 | urine | France | 2015 |
| PRJNA551371 | SRP212396 | SRS5039637 | SRX6378983 | SRR9616023 | 405 | urine | France | 2016 |
| PRJNA551371 | SRP212396 | SRS5039621 | SRX6378967 | SRR9616039 | 405 | urine | France | 2016 |
| PRJEB21201 | ERP023438 | ERS1792259 | ERX2070645 | ERR2010973 | 155 | - | Bangladesh | 2015 |
| PRJEB21201 | ERP023438 | ERS1792192 | ERX2070578 | ERR2010906 | 155 | - | Bangladesh | 2015 |
| PRJEB39855 | ERP123420 | ERS4944679 | ERX4396445 | ERR4456074 | 448 | stool | Bangladesh | 2007 |
| PRJNA607273 | SRP252199 | SRS6296916 | SRX7887286 | SRR11281076 | 155 | stool | Bangladesh | missing |
| PRJNA607273 | SRP252199 | SRS6296783 | SRX7887153 | SRR11280865 | 155 | stool | Bangladesh | missing |
| PRJNA607273 | SRP252199 | SRS6296780 | SRX7887151 | SRR11280867 | 155 | stool | Bangladesh | missing |
| PRJNA607273 | SRP252199 | SRS6296777 | SRX7887147 | SRR11280871 | 155 | stool | Bangladesh | missing |
| PRJNA607273 | SRP252199 | SRS6296723 | SRX7887093 | SRR11280925 | 155 | stool | Bangladesh | missing |
| PRJNA611810 | SRP264772 | SRS6711837 | SRX8399055 | SRR11848768 | 131 | stool | Bangladesh | missing |
| PRJNA611810 | SRP264772 | SRS6711826 | SRX8399044 | SRR11848779 | 155 | stool | Bangladesh | missing |
| PRJNA611810 | SRP264772 | SRS6711816 | SRX8399034 | SRR11848789 | 38 | stool | Bangladesh | missing |
| PRJNA762607 | SRP337282 | SRS10176422 | SRX12200081 | SRR15909664 | 131 | urine | Bangladesh | missing |
| PRJNA762607 | SRP337282 | SRS10176421 | SRX12200080 | SRR15909665 | 38 | urine | Bangladesh | missing |
| PRJNA762607 | SRP337282 | SRS10176416 | SRX12200075 | SRR15909670 | 155 | urine | Bangladesh | missing |
| PRJEB56918 | ERP141886 | ERS13615940 | ERX9966608 | ERR10441080 | 131 | urine | Bangladesh | 2020 |
| PRJEB56918 | ERP141886 | ERS13615946 | ERX9966606 | ERR10441078 | 131 | urine | Bangladesh | 2020 |
| PRJNA1013156 | SRP458677 | SRS18809623 | SRX21639717 | SRR25920017 | 405 | urine | Bangladesh | 2022 |
| PRJNA1013156 | SRP458677 | SRS18809620 | SRX21639716 | SRR25920018 | 38 | wound | Bangladesh | 2022 |

**Table S2**. List of antibiotic resistance genes identified in ESBL-Ec isolates obtained from three different sources.

| Antibiotic class | Gene | Poultry (n=12) | Human (n=20) | Environment (n=85) |
| --- | --- | --- | --- | --- |
| Aminoglycosides |  |  |  |  |
|  | *aac(3)-IIa* | 2 | 1 | 11 |
|  | *rmtB* | 1 | 1 | 24 |
|  | *aadA2* | 4 | 5 | 33 |
|  | *aadA5* | 3 | 1 | 13 |
|  | *ant(3'')-Ia* | 4 | 1 | 15 |
|  | *aph(3'')-Ib* | 2 | 1 | 8 |
|  | *aph(6)-Id* | 2 | 1 | 11 |
| Beta-lactam |  |  |  |  |
|  | *bla*_CTX-M-15_ | 6 | 19 | 64 |
|  | *bla*_OXA-1_ | 2 | 4 | 24 |
|  | *bla*_TEM-1B_ | 9 | 6 | 51 |
| Carbapenem |  |  |  |  |
|  | *bla*_NDM-5_ | 1 | 3 | 30 |
| Macrolide |  |  |  |  |
|  | *erm(B)* | 1 | 3 | 17 |
|  | *mph(A)* | 6 | 8 | 38 |
| Phenicol |  |  |  |  |
|  | *catA1* | 1 | 1 | 7 |
|  | *catB3* | 2 | 4 | 24 |
| Fluoroquinolone |  |  |  |  |
|  | *aac(6')-Ib-cr* | 2 | 4 | 23 |
| Quinolone |  |  |  |  |
|  | *qnrS1* | 4 | 13 | 33 |
| Sulphonimide |  |  |  |  |
|  | *sul1* | 4 | 5 | 40 |
|  | *sul2* | 3 | 1 | 9 |
| Tetracycline |  |  |  |  |
|  | *tet(A)* | 8 | 5 | 30 |
|  | *tet(B)* | 1 | 2 | 24 |
| Trimethoprim |  |  |  |  |
|  | *dfrA1* | 1 | 1 | 8 |
|  | *dfrA12* | 4 | 5 | 33 |
|  | *dfrA14* | 1 | 1 | 4 |
|  | *dfrA17* | 3 | 1 | 13 |

**Table S3**. Number of resistance genes identified in ESBL-Ec obtained from three different sources.

| Antibiotic class | Gene | Poultry (n=12) | Human (n=20) | Environment (n=85) |
| --- | --- | --- | --- | --- |
| Aminoglycosides |  |  |  |  |
|  | *aac*(3)-IIa | 2 | 1 | 11 |
|  | *aac*(3)-IId | 1 | 0 | 6 |
|  | *aac*(3)-IVa | 2 | 0 | 0 |
|  | *aac*(6')-Ib-Hangzhou | 0 | 0 | 1 |
|  | *rmt*B | 1 | 1 | 24 |
|  | *rmt*C | 0 | 0 | 3 |
|  | *aad*A2 | 4 | 5 | 33 |
|  | *aad*A5 | 3 | 1 | 13 |
|  | *ant*(3'')-Ia | 4 | 1 | 15 |
|  | *aph*(3'')-Ib | 2 | 1 | 8 |
|  | *aph*(3')-Ia | 2 | 0 | 8 |
|  | *aph*(4)-Ia | 2 | 0 | 0 |
|  | *aph*(6)-Id | 2 | 1 | 11 |
|  | *arm*A | 0 | 0 | 1 |
| Beta-lactam |  |  |  |  |
|  | *bla*_CMY-141_ | 0 | 0 | 2 |
|  | *bla*_CMY-145_ | 0 | 0 | 2 |
|  | *bla*_CMY-146_ | 0 | 1 | 0 |
|  | *bla*_CMY-2_ | 0 | 0 | 2 |
|  | *bla*_CMY-42_ | 0 | 0 | 9 |
|  | *bla*_CTX-M-123_ | 1 | 0 | 0 |
|  | *bla*_CTX-M-14_ | 0 | 1 | 0 |
|  | *bla*_CTX-M-15_ | 6 | 19 | 64 |
|  | *bla*_CTX-M-55_ | 1 | 0 | 5 |
|  | *bla*_CTX-M-64_ | 0 | 0 | 1 |
|  | *bla*_CTX-M-65_ | 3 | 0 | 0 |
|  | *bla*_DHA-1_ | 0 | 0 | 6 |
|  | *bla*_DHA-24_ | 0 | 0 | 3 |
|  | *bla*_DHA-7_ | 0 | 0 | 3 |
|  | *bla*_OXA-1_ | 2 | 4 | 24 |
|  | *bla*_OXA-10_ | 0 | 0 | 1 |
|  | *bla*_OXA-181_ | 0 | 0 | 2 |
|  | *bla*_OXA-320_ | 0 | 0 | 1 |
|  | *bla*_OXA-534_ | 0 | 0 | 1 |
|  | *bla*_OXA-9_ | 0 | 0 | 1 |
|  | *bla*_SHV-187_ | 1 | 0 | 0 |
|  | *bla*_TEM-104_ | 0 | 0 | 4 |
|  | *bla*_TEM-141_ | 1 | 0 | 3 |
|  | *bla*_TEM-164_ | 0 | 0 | 1 |
|  | *bla*_TEM-198_ | 0 | 0 | 1 |
|  | *bla*_TEM-1B_ | 9 | 6 | 51 |
|  | *bla*_TEM-206_ | 1 | 0 | 4 |
|  | *bla*_TEM-209_ | 1 | 0 | 2 |
|  | *bla*_TEM-210_ | 1 | 0 | 0 |
|  | *bla*_TEM-214_ | 1 | 0 | 4 |
|  | *bla*_TEM-216_ | 1 | 0 | 0 |
|  | *bla*_TEM-33_ | 1 | 0 | 0 |
|  | *bla*_TEM-34_ | 1 | 0 | 0 |
| Carbapenem |  |  |  |  |
|  | *bla*_NDM-1_ | 0 | 0 | 4 |
|  | *bla*_NDM-5_ | 1 | 3 | 30 |
|  | *bla*_NDM-7_ | 0 | 1 | 6 |
| Colistin |  |  |  |  |
|  | *mcr*-1.1 | 0 | 0 | 1 |
|  | *mcr*-9 | 0 | 0 | 3 |
| Fosfomycin |  |  |  |  |
|  | *fos*A3 | 5 | 6 | 40 |
| Macrolide |  |  |  |  |
|  | *erm*(B) | 5 | 5 | 19 |
|  | *mef*(B) | 0 | 0 | 2 |
|  | *mph*(A) | 6 | 8 | 38 |
|  | *mph*(E) | 0 | 0 | 6 |
|  | *msr*(E) | 0 | 0 | 6 |
| Phenicol |  |  |  |  |
|  | *cat*A1 | 1 | 1 | 7 |
|  | *cat*A2 | 0 | 0 | 1 |
|  | *cat*B3 | 2 | 4 | 24 |
|  | *cml*A1 | 3 | 0 | 9 |
|  | *flo*R | 1 | 0 | 1 |
| Fluoroquinolone |  |  |  |  |
|  | *aac*(6')-Ib-cr | 2 | 4 | 23 |
| Quinolone |  |  |  |  |
|  | *qep*A4 | 0 | 0 | 4 |
|  | *qnr*B1 | 0 | 0 | 1 |
|  | *qnr*B2 | 0 | 0 | 1 |
|  | *qnr*B4 | 0 | 0 | 3 |
|  | *qnr*S1 | 4 | 13 | 33 |
|  | *qnr*S13 | 1 | 0 | 7 |
|  | *qnr*S4 | 1 | 0 | 1 |
|  | *qnr*VC1 | 0 | 1 | 0 |
| Rifampicin |  |  |  |  |
|  | *ARR*-3 | 0 | 0 | 4 |
| Sulphonimide |  |  |  |  |
|  | *sul*1 | 4 | 5 | 40 |
|  | *sul*2 | 3 | 1 | 9 |
|  | *sul*3 | 4 | 0 | 9 |
| Tetracycline |  |  |  |  |
|  | *tet*(A) | 8 | 5 | 30 |
|  | *tet*(B) | 1 | 2 | 24 |
| Trimethoprim |  |  |  |  |
|  | *dfr*A1 | 1 | 1 | 8 |
|  | *dfr*A12 | 4 | 5 | 33 |
|  | *dfr*A14 | 1 | 1 | 4 |
|  | *dfr*A15 | 1 | 0 | 2 |
|  | *dfr*A17 | 3 | 1 | 13 |
|  | *dfr*A23 | 0 | 0 | 3 |
|  | *dfr*A5 | 0 | 0 | 1 |
|  | *dfr*A7 | 0 | 0 | 1 |

**Table S4**. Clermont phylotype and Achtman MLST classification for ESBL-Ec isolates from poultry, human, and environmental sources. Total percent is out of all 117 isolates.

| Phylotypes | ST (n) | Poultry (n=12) | Human (n=20) | Environment (n=85) | Total (%) |
| --- | --- | --- | --- | --- | --- |
| A | All | 4 | 7 | 20 | 26.50 |
|  | 10 | 0 | 1 | 2 | 2.56 |
|  | 1072 | 0 | 0 | 1 | 0.85 |
|  | 12452 | 0 | 0 | 1 | 0.85 |
|  | 13212 | 0 | 0 | 1 | 0.85 |
|  | 1408 | 1 | 0 | 0 | 0.85 |
|  | 167 | 0 | 0 | 1 | 0.85 |
|  | 1818 | 0 | 0 | 1 | 0.85 |
|  | 206 | 1 | 1 | 0 | 1.71 |
|  | 226 | 0 | 1 | 1 | 1.71 |
|  | 2935 | 0 | 0 | 1 | 0.85 |
|  | 3489 | 0 | 1 | 3 | 3.42 |
|  | 361 | 0 | 0 | 2 | 1.71 |
|  | 4450 | 0 | 0 | 2 | 1.71 |
|  | 46 | 0 | 0 | 1 | 0.85 |
|  | 48 | 1 | 0 | 1 | 1.71 |
|  | 50 | 0 | 1 | 0 | 0.85 |
|  | 542 | 0 | 1 | 0 | 0.85 |
|  | 746 | 0 | 0 | 1 | 0.85 |
|  | 752 | 1 | 0 | 0 | 0.85 |
|  | 757 | 0 | 0 | 1 | 0.85 |
|  | 93 | 0 | 1 | 0 | 0.85 |
| B1 | All | 5 | 8 | 32 | 38.46 |
|  | 101 | 0 | 0 | 4 | 3.42 |
|  | 1196 | 1 | 0 | 0 | 0.85 |
|  | 13214 | 0 | 0 | 1 | 0.85 |
|  | 155 | 3 | 2 | 8 | 11.11 |
|  | 156 | 0 | 0 | 1 | 0.85 |
|  | 1727 | 0 | 0 | 3 | 2.56 |
|  | 205 | 0 | 1 | 1 | 1.71 |
|  | 2852 | 0 | 0 | 1 | 0.85 |
|  | 448 | 0 | 1 | 5 | 5.13 |
|  | 4684 | 0 | 1 | 0 | 0.85 |
|  | 515 | 0 | 0 | 1 | 0.85 |
|  | 517 | 0 | 0 | 1 | 0.85 |
|  | 5869 | 1 | 0 | 0 | 0.85 |
|  | 602 | 0 | 1 | 1 | 1.71 |
|  | 6683 | 0 | 1 | 1 | 1.71 |
|  | 683 | 0 | 0 | 1 | 0.85 |
|  | 711 | 0 | 1 | 0 | 0.85 |
|  | 8346 | 0 | 0 | 3 | 2.56 |
| B2 | All | 0 | 1 | 1 | 1.71 |
|  | 131 | 0 | 1 | 1 | 1.71 |
| C | All | 0 | 0 | 5 | 4.27 |
|  | 2851 | 0 | 0 | 1 | 0.85 |
|  | 410 | 0 | 0 | 3 | 2.56 |
|  | 90 | 0 | 0 | 1 | 0.85 |
| D | All | 2 | 2 | 22 | 22.22 |
|  | 1011 | 1 | 0 | 0 | 0.85 |
|  | 1588 | 0 | 0 | 2 | 1.71 |
|  | 315 | 0 | 0 | 1 | 0.85 |
|  | 38 | 0 | 0 | 5 | 4.27 |
|  | 405 | 1 | 0 | 13 | 11.97 |
|  | 68 | 0 | 1 | 1 | 1.71 |
|  | 973 | 0 | 1 | 0 | 0.85 |
| E | All | 0 | 0 | 1 | 0.85 |
|  | 1140 | 0 | 0 | 1 | 0.85 |
| F | All | 1 | 2 | 3 | 5.13 |
|  | 13213 | 1 | 0 | 0 | 0.85 |
|  | 13215 | 0 | 1 | 0 | 0.85 |
|  | 1722 | 0 | 0 | 1 | 0.85 |
|  | 2141 | 0 | 0 | 1 | 0.85 |
|  | 648 | 0 | 1 | 1 | 1.71 |
| G | All | 0 | 0 | 1 | 0.85 |
|  | 1163 | 0 | 0 | 1 | 0.85 |

**Table S5**. Antimicrobial resistance (AMR) gene profiles of plasmids recovered from long read sequencing data for two carbapenem-resistant isolates.

| **Isolate ID** | **Source** | **Plasmid type** | **Plasmid AMR genes (CARD protein id/coverage ≥90%)** |
| --- | --- | --- | --- |
| DL-185-SS2-K1 | Solid waste sample from an urban wet market | IncFIA | macrolide: *mrx* (note: incomplete multi-component inactivation cluster *mphA*-*mrx*-*mphR*)  sulfonamide: *sul1*  aminoglycoside: *aadA5*  phenicol: *catA1*  tetracycline: *tetR*, *tetB*  biocide: *qacE-delta 1* |
|  |  | IncFII(29) | broad spectrum beta-lactam: *bla*_OXA-1_*, bla*_TEM-1_  extended spectrum beta-lactam: *bla*_NDM-5_  sulfonamide: *sul1*  trimethoprim: *dfrA12*  phenicol: *catB3*  aminoglycoside: *aac(6’)-Ib-cr6*, *aadA2,* *rmtB*  biocide: *qacE-delta 1* |
|  |  | IncY | None identified |
| TR_110_DRW-K1 | Downstream river water from a rural area | IncH | aminoglycoside: *aph(3’-I), aac(3)-IId, aadA2*  tetracycline: *tetM*  broad spectrum beta-lactam: *bla*_TEM-1_  sulfonamide: *sul3*  colistin: *mcr 1.1*  phenicol: *cmlA1*  biocide: *qacL* |
|  |  | IncX | broad spectrum beta-lactam: *bla*_TEM-1_  sulfonamide: *sul3*  trimethoprim: *dfrA14*  tetracycline: *tetA* |

**Figure Ledgends**

**Figure S1**. Figure depicting the presence of resistance and virulence genes with plasmid types in terms of whether or not a gene was present for the top 16 resistance genes, top 15 virulence genes, and all plasmid types detected. Black squares represent the absence, and green represents the presence of each sample’s resistance class. Red represents the presence of a plasmid. Source niche (poultry, human, environment) is represented by orange, purple, and yellow, respectively.

**Figure S2**. Violin plot comparing the ARG diversity among wastewater isolates obtained from three different study sites including urban wet markets, rural households, and rural poultry farms through Kruskal-Wallis test and Dunn pairwise test.

**Figure S3**. Violin plot comparing the total resistance genes between two groups of samples: those without plasmids (teal) and those with plasmids (orange). The plot displays the distribution of resistance genes within each group, illustrating a notable difference in the total resistance genes between the two groups. A Welch's t-test was conducted, revealing a statistically significant difference between the groups with a p-value of <0.001. The presence of plasmids is associated with a higher number of resistance genes.

**Figure S4**. Grapetree displaying a MSTree v2 generated using cgMLST V1 + HierCC V1. Node ID indicates study sites such as wet markets, rural households, and rural poultry farms (LM, HH, PF, respectively). Colors indicating location LM (grey), HH (pink), PF (brown) and clinical (red). Distance refers to the number of differing alleles. Combined nodes are <11 alleles different.
